# Deciphering HIV-1 Transcription Initiation and Elongation from Single-Molecule Imaging Data

**DOI:** 10.34133/research.0645

**Published:** 2025-03-31

**Authors:** Xiyan Yang, Zihao Wang, Changhong Shi, Tianshou Zhou, Jiajun Zhang

**Affiliations:** ^1^ School of Financial Mathematics and Statistics, Guangdong University of Finance, Guangzhou 510521, P. R. China.; ^2^ Guangdong Province Key Laboratory of Computational Science, Sun Yat-sen University, Guangzhou 510275, P. R. China.; ^3^ School of Mathematics, Sun Yat-sen University, Guangzhou 510275, P. R. China.; ^4^ The State Key Laboratory of Respiratory Disease, School of Public Health, Guangzhou Medical University, Guangzhou 510182, P. R. China.

## Abstract

The stages of transcription initiation and elongation are critical in the regulation of HIV-1 gene expression. Recent single-molecule imaging in living cells has shown that HIV-1 transcription occurs across multiple time scales and plays a key role in the control of latency. However, the molecular mechanisms of HIV-1 transcription remain poorly understood due to the lack of a unified modeling framework and advanced computational methods for analyzing HIV-1 imaging data. Here, we present a general stochastic model that characterizes HIV-1 transcription dynamics and computes the distributions of initiation times and nascent RNA counts. Our results show that coordination between initiation and elongation modulates transcription dynamics and that leveraging initiation-time data enhances model identification. Meanwhile, we develop a statistical inference method that integrates initiation-time data and nascent RNA data. Our results show that incorporating initiation-time data allows for accurate inference of the initiation rate and elongation time, with these parameter estimates being independent of the models used. When applied to HIV-1 transcription data in living cells, our theory and inference methods confirm the dual role of Tat in HIV-1 transcriptional regulation. In addition, the optimal predictive model indicates that Tat induces viral reactivation and latency exit by altering the number of silent states of the promoter. Our approach may provide the potential to improve current HIV-1 cure strategies.

## Introduction

Transcription is a complex process that involves 2 key stages, initiation and elongation, each playing an important role in the regulation of gene expression [1–4]. Studies have shown that initiation and elongation coordinately control gene expression across living organisms [5–7]. For example, stimulating transcription initiation contributes to the activation of latent human immunodeficiency virus–1 (HIV-1) proviruses, while blocking transcription elongation helps establish and maintain proviral latency [8,9]. Both of these processes are crucial for understanding the maintenance and reactivation of the virus and for developing effective strategies to modulate latent viruses toward a functional cure. However, the precise molecular mechanisms through which transcription initiation and elongation coordinate gene expression remain unclear.

Addressing these questions requires experimental measurements of nascent RNA transcription. Recent advances in sequencing techniques have enabled the detection of initiation or elongation rates on a gene-by-gene or genome-wide basis, facilitating the identification of the key molecular components of transcription and their interactions [10–13]. Single-molecule fluorescence in situ hybridization (FISH) allows the measurement of these kinetic rates by counting nascent RNAs in thousands of fixed single cells, providing valuable insights into transcriptional regulation [14,15]. However, genome-wide and single-molecule FISH methods have existing limitations, including low resolution [16,17], high noise [18,19], and measuring relative rather than absolute rates of initiation or elongation [20–22]. Furthermore, these detection techniques lack the temporal resolution required to analyze dynamic cellular processes. Recently developed live-cell imaging techniques enable direct detection of in vivo time-resolved RNA fluorescence for different genes [23–25], making it possible to analyze the dynamic behaviors of transcription initiation and elongation from imaging data. To fully realize the potential of these imaging techniques, it will be crucial to develop mathematical models and computational methods to measure kinetic parameters for dissecting the molecular mechanisms of transcriptional regulation.

Stochastic models, including the 2-state model [22,26–29] and multistate models [16,30–32], have been widely used for the analysis of FISH or sequencing data. In these models, transcription dynamics are inferred by fitting the steady-state distribution of nascent RNA or messenger RNA counts. However, parameter estimates from the steady-state distribution of stochastic models are often normalized, with the assumption that one or more parameters are fixed or estimated in advance from other experiments [26,28,33,34]. Although normalized methods facilitate analysis, it is difficult to obtain accurate absolute parameter values due to the loss of temporal information, which is crucial for a comprehensive understanding of the true dynamics of gene regulatory systems. Recently, several novel studies have combined simple stochastic models with initiation-time data in live cells to explore transcriptional dynamics [35–37]. For example, the occurrence of multiple time scales of transcriptional bursting has been directly detected from the measurements of HIV-1 promoter activity using initiation-time data from the MS2 system and simple stochastic models [38,39]. However, analyses based on initiation-time data do not take into account the subsequent processes, such as elongation, which play a crucial role in regulating transcription [40–42].

In fact, gene expression is regulated in a coordinated manner by the stochastic timing of transcription initiation, which integrates multiple biochemical processes, and by the number of nascent RNA molecules controlled during elongation [24,43–45]. A major challenge is to develop mathematical models that can comprehensively analyze the dynamics of transcription initiation and elongation. In principle, the models constructed should satisfy some basic requirements. First, these models should be interpretable; i.e., they should provide a mechanistic understanding of transcription dynamics. Previous studies have employed both deterministic kinetic models [46,47] and simple stochastic models [48–50], but neither of these approaches is sufficient for characterizing HIV-1 transcription across multiple time scales. Therefore, a unified model that recapitulates the full range of HIV-1 promoter states is required. Second, the models constructed should be tractable; i.e., they should not only handle static gene expression data but also decode temporal information. In general, complex models incorporating feedback regulation often present substantial mathematical challenges [51–53] and pose considerable difficulties in inferring HIV-1 kinetic parameters from imaging data. Furthermore, despite the use of nascent RNA data and initiation-time data, these are often used in isolation to estimate transcriptional kinetic parameters as discussed above. A further challenge is to develop computational methods that integrate these 2 types of data to infer transcription initiation and elongation dynamics simultaneously. Therefore, both an interpretable and tractable mathematical model and an efficient inference framework that integrates nascent RNA data and initiation data from single-molecule imaging are strongly demanded.

In this study, we propose a general theoretical framework that incorporates the gene promoter, initiation, and elongation processes to analyze the transcription dynamics of HIV-1. This framework allows the derivation of 2 analytical distributions of initiation times and nascent RNA counts. We then use these analytical results to explore how initiation and elongation coordinate to modulate transcription dynamics over large parameter spaces. Building on this theory, we develop a statistical inference method that integrates nascent RNA data and initiation-time data to estimate the transcription dynamics of HIV-1. Our results show that when inference is performed using the 2-state model with the addition of initiation-time data, the true initiation rate and elongation time can be accurately estimated without fixing any other parameters, regardless of the models generating the data. Furthermore, our results indicate that, in addition to the 2-state model, multistate models can also accurately estimate these 2 transcriptional parameters, implying that the estimates of these 2 parameters are model independent. Our methods were then applied to the live-cell HIV-1 transcription imaging data, providing new evidence for a long-standing debate regarding the dual role of Tat in viral transcription [8,54]. Our results also indicate that the maintenance of latency requires more silent promoter states compared to high Tat expression. Overall, our unified model and data-integrated inference methods provide an ideal framework for quantifying HIV-1 transcriptional dynamics in live cells.

## Results

### A general HIV-1 stochastic transcription model

**Fig. 1.**
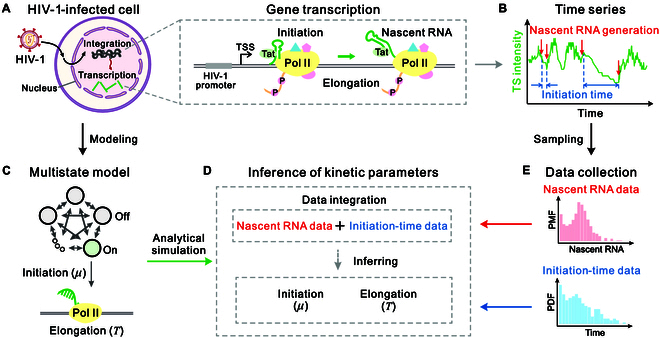
Construction of a general HIV-1 transcription model to infer transcription dynamics from time series data. (A) Schematic of gene transcription in HIV-1 infected cells. This complex process of HIV-1 transcription involves 2 critical phases: the initiation phase, where the transcription complex such as RNA polymerase II (Pol II), interacts with the viral factor Tat to trigger transcription, and the elongation phase, where the RNA transcript is synthesized to drive the viral replication cycle. (B) Illustration of time series data obtained from RNA imaging of the nucleus, where both nascent RNA counts quantified by transcription site (TS) intensity and transcription initiation events can be detected. (C) The complex process of HIV-1 transcription in (A) is mapped to a multistate model that includes multiple inactive (off) states and one active (on) state. (D) Schematic of the inference of HIV-1 transcriptional parameters based on the integration of nascent RNA data and initiation-time data. (E) Illustration of nascent RNA distribution and initiation-time distribution for performing inference, with data obtained from sampling time series data. TSS, transcription start site; PMF, probability mass function; PDF, probability density function.

The transcription of HIV-1 is a complex and dynamic process that involves a multitude of regulatory interactions, including the binding of transcription factors to the viral promoter, initiation of transcription, and elongation of the nascent RNA (Fig. 1A). A key player in this process is the viral Tat protein, which strongly enhances viral transcription in acutely infected cells by interacting with the *cis*-acting RNA element trans-activation response (TAR) at the long terminal repeat promoter [55,56]. Conversely, in transcriptionally silenced proviral cells, the absence of Tat is regarded as a major barrier to the reactivation of viral expression [57]. HIV-1 transcription can be observed in living cells using the MS2 and MS2 coat protein (MS2–MCP) system [39], with imaging data containing information on both nascent RNA levels and transcription initiation events (Fig. 1B).

Studies have shown that HIV-1 transcription is discontinuous and undergoes alternative periods of activity and inactivity in a stochastic manner [48,51,58]. To elucidate the fundamental dynamics of transcriptional regulation in HIV-1, we construct a multistate model for analyzing time series data (Fig. 1C). In this model, the viral promoter contains multiple inactive (off) states and an active (on) state, and these states can switch arbitrarily with each other. We introduce such a multistate model based on the following 2 facts: (a) The complex transcription process in HIV-1 involving the recruitment of host polymerases, the assembly of pre-initiation complexes, interactions with viral regulatory proteins, and chromatin remodeling, cannot be adequately described by single-state or 2-state promoter switching [8,59,60]. (b) Recent experimental evidence has shown that the prolonged latent state in HIV-1 gene expression is associated with stochastic inactivation of the gene, implying that the promoter may exist in multiple off states [39,49,61]. In particular, we consider a transcription model with a loop promoter structure; i.e., the promoter proceeds sequentially through several irreversible states with multiple inactive (off) states and one active (on) state, which together form a loop. Note that the multistate loop model reduces to the classical 2-state model in the case of a single off state.

To analyze the transcription initiation events from time series data using the multistate model, we first need to determine the distribution of initiation times. Assume that the promoter switches among N different states with transition rates $\lambda_{\mathrm{kl}}$ from the kth state to the lth state, and denote the transition matrix $A=\left(\lambda_{\mathrm{kl}}\right)$ ($\lambda_{\mathrm{kl}}=0$ means that no transition takes place). In addition, we define the transcription matrix $\mu =\text{diag}\left(0,0,\cdots ,\mu \right)$. Let $P_{k}\left(t\right)$ represent the probability that the promoter is state k at time t, given that no initiation event occurs between 0 and t. Let $P\left(t\right)={\left(P_{1}\left(t\right),P_{2}\left(t\right),\cdots ,P_{N}\left(t\right)\right)}^{T}$ represent the column vector. The initiation matrix is denoted by $A_{\mathrm{ini}}={\left(A-\mu \right)}^{T}$ the initiation matrix, where the superscript T denotes matrix transposition. Then, the master equation is given by

$$\frac{dP\left(t\right)}{\mathrm{dt}}=A_{\mathrm{ini}}P\left(t\right)$$ (1)

Solving the master equation yields $P\left(t\right)=e^{A_{\mathrm{ini}}t}P\left(0\right)$ with the initial condition $P\left(0\right)={\left(P_{1}\left(0\right),P_{2}\left(0\right),\cdots ,P_{N}\left(0\right)\right)}^{T}$. Define an N-dimensional row vector $u_{N}=\left(1,1,\cdots ,1\right)$; then, the probability density function (PDF) of the initiation-time distribution is given by

$$f_{\mathrm{ini}}\left(t\right)=u_{\mathrm{ini}}P\left(t\right)=u_{\mathrm{ini}}e^{A_{\mathrm{ini}}t}P\left(0\right)$$ (2)

where $u_{\mathrm{ini}}=u_{N}\mu$ is an N-dimensional row vector. The Laplace transform of $f_{\mathrm{ini}}\left(t\right)$ can be given by ${\tilde{f}}_{\mathrm{ini}}\left(\;s\;\right)=u_{\mathrm{ini}}{\left(\;sI-A_{\mathrm{ini}}\;\right)}^{-1}P\left(0\right)$, where I is the N×N identity matrix, which can be further written as a rational function. By performing the inverse Laplace transform on this rational form, we can derive the analytical expression for the PDF of the initiation-time distribution (refer to Eq. 5 in Methods). Unlike the exponential and mixed exponential distributions, this expression encompasses multiple forms, allowing for the analysis of transcription initiation events in HIV-1 expression across a range of time scales.

However, the distribution of initiation times alone cannot fully characterize transcription kinetics, as it does not take into account subsequent processes such as elongation. In fact, nascent RNA elongation plays a critical role in the HIV-1 life cycle, directly affecting the replication efficiency and infectivity of the virus [8,55]. Thus, it is necessary to consider the distribution of nascent RNA counts, which integrates these subsequent processes [22,62,63]. Assume that the elongation of a single transcript is deterministic and that the elongation time is T. A transcription elongation process may be very complex, involving pausing and backtracking of polymerases along the gene body. Nevertheless, our assumption of deterministic elongation is justified for many genes [24,27,64,65]. In the following, we derive the nascent RNA distribution based on the transcription initiation events for the multistate model.

Let $T_{m}$ denote the waiting time between the mth and $\left(m-1\right)\mathrm{th}$ nascent RNA production events and $t_{m}=\sum_{j=1}^{m}T_{j}$ denote the time of the mth nascent RNA production event. Then, the number of nascent RNA production events $M\left(t\right)$ that occurred at $t_{m}\leq t<t_{m+1}$ constitutes a renewal process [66]. Our interest is to find the steady-state distribution of the number of nascent RNAs for t>T. Denote $f_{1}\left(t\right)$ as the PDF of the time for the first initiation event occurrence; then, the steady-state nascent RNA distribution $P\left(M=m\right)$ based on the initiation-time distribution has the following form (see Text S3 for details):

$$\begin{array}{c} P\left(M=m\right)=\left\{\begin{array}{cc} \;\int_{T}^{\infty }f_{1}\left(\tau \right)\mathrm{d\tau}, & m=0,\\ \left(f_{1}*f_{\mathrm{ini}}^{m-1}*S_{\mathrm{ini}}\right)\left(T\right), & m\geq 1, \end{array}\right. \end{array}$$ (3)

where $S_{\mathrm{ini}}\left(t\right)=\int_{t}^{\infty }f_{\mathrm{ini}}\left(\tau \right)\;\mathrm{d\tau}$ is the survival function of $f_{\mathrm{ini}}\left(t\right)$ and $f_{\mathrm{ini}}^{m-1}\left(t\right)$ is the m−1-fold convolution of $f_{\mathrm{ini}}\left(t\right)$. By straightforward calculation, we further derive the analytical expressions of the steady-state nascent RNA distribution (refer to Eq. 6 in Methods). The analytical distributions of nascent RNA counts and initiation times are crucial for quantitatively characterizing the transcription dynamics of HIV-1.

To analyze transcription dynamics from time series data, it is essential to collect both nascent RNA data and initiation-time data to calculate the corresponding distributions (Fig. 1E). The nascent RNA data can be obtained through uniform sampling, while the initiation-time data can be acquired by recording the time intervals between consecutive transcription initiation events. The aim of this study is to apply the analytical results obtained from the constructed model and combine nascent RNA data with initiation-time data to infer the transcription dynamics of HIV-1. To achieve this, we propose a data-integrated inference method. Specifically, we integrate nascent RNA data and initiation-time data to infer transcription kinetic parameters using maximum likelihood estimation (MLE), based on the joint distribution of these 2 types of data (Fig. 1D). The inference can be formulated as the following optimization problem:

$$\begin{array}{c} \underset{\theta }{\text{arg}}\text{min}\left(-L\left(\theta \right)\right)=\\ \underset{\theta }{\text{arg}}\text{min}\left(-\left(\sum_{i}\text{ln}f_{\mathrm{ini}}\left(\tau_{i};\;\theta \right)+\sum_{j}\text{ln}P\left(m_{j};\;\theta \right)\right)\right) \end{array}$$ (4)

where $L\left(\theta \right)=\prod_{i,j}P\left(\tau_{i},\;m_{j};\;\theta \right)$ is the total likelihood function with $P\left(\tau_{i},\;m_{j};\;\theta \right)$ being the joint distribution; $\tau_{i}$ and $m_{j}$ are the ith initiation-time data and jth nascent RNA data, respectively, both of which are collected from the time series data; and $\theta =\left(\lambda_{\mathrm{kl}},\;\mu ,\;T\right)$ is the parameter vector of the multistate model to be estimated (see Methods).

### Leveraging initiation-time data for enhanced model identification

In this section, we explore how initiation and elongation coordinate to regulate transcription. Although the study of transcription dynamics using static expression data has become a widely adopted approach, a major challenge associated with this method is the unidentifiability of the model. For example, different stochastic models can lead to the same steady-state distribution [32,67], making it difficult to accurately determine the underlying biological processes. Therefore, incorporating initiation-time data is particularly important for understanding transcription regulatory mechanisms.

Based on the theory described above, we explore the stochastic bifurcation phase diagrams for the distributions of initiation times and nascent RNA counts across a large region of parameter space. For convenience, we consider a multistate loop model (5-state) and assume that the transition rates among the off states, as well as the transition rate from the last off state to the on state, are identical and denoted by $k_{f}$ (called the forward transition rate), while the transition rate from the on state to the first off state is denoted by $k_{b}$ (called the backward transition rate). We adjust the 2 transition rates $k_{f}$ and $k_{b}$ to calculate the distributions of initiation times and nascent RNA counts. Since the exact distribution of nascent RNA involves factorials and powers, direct calculations can be computationally expensive. Therefore, we use the finite state projection method to efficiently solve the multistate model (Fig. S1) [68].

**Fig. 2.**
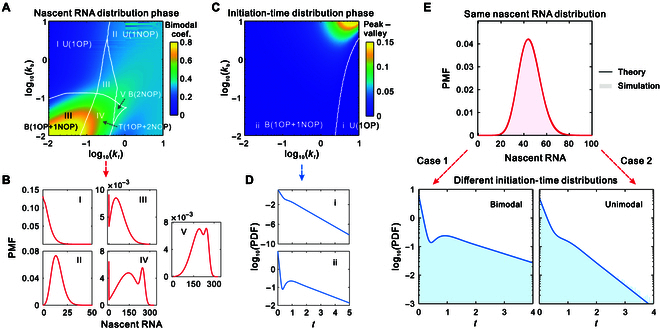
Effects of initiation times on model identification. (A) A stochastic bifurcation diagram for the steady-state nascent RNA distribution as a function of the forward transition rate $k_{f}$ and the backward transition rate $k_{b}$. The white curves classify the unimodal region with an origin peak (denoted by U(1OP), region I), unimodal region with a non-origin peak (denoted by U(1NOP), region II), bimodal region with an origin peak and a non-origin peak (denoted by B(1OP+1NOP), region III), trimodal region with an origin peak and 2 non-origin peaks (denoted by T(1OP+2NOP), region IV), and bimodal region with 2 non-origin peaks (denoted by B(2NOP), region V). (B) Five representative PMFs for the 5 regions. (C) A stochastic bifurcation diagram for the initiation-time distribution as a function of the forward transition rate $k_{f}$ and the backward transition rate $k_{b}$. The white curve classifies the unimodal region with an origin peak (denoted by U(1OP), region i) and bimodal region with an origin peak and a non-origin peak (denoted by B(1OP+1NOP), region ii). (D) Two PDFs for the 2 regions. (E) Two same nascent RNA distributions from different model parameters exhibit different initiation-time distributions, where solid curves are theoretical predictions and the filled areas represent the SSA [69]. Parameter values are set as μ=10 and T=25 in (A) and (C) and $k_{f}=4.3633$, $k_{b}=5.1358$, μ=6.2109, and T=41.4842 for a bimodal PDF and $k_{f}=6$, $k_{b}=1$, μ=5, and T=15 for a unimodal PDF in (E).

We first explore the shapes of the steady-state nascent RNA distribution and identify 5 distinct regions (Fig. 2A): a unimodal region with an origin peak (denoted by U(1OP)), a unimodal region with a non-origin peak (denoted by U(1NOP)), a bimodal region with both an origin peak and a non-origin peak (denoted by B(1OP+1NOP)), a trimodal region with an origin peak as well as 2 non-origin peaks (denoted by T(1OP+2NOP)), and a bimodal region with both non-origin peaks (denoted by B(2NOP)). Furthermore, the heatmap in Fig. 2A represents the bimodal coefficient (see Methods), which is often taken as a measure of the modality of a distribution [63]. Figure 2B illustrates 5 representative steady-state distributions of nascent RNA for the 5 regions. Next, we explore the shapes of the initiation-time distribution within the same parameter space as the nascent RNA distribution and identify 2 distinct regions (Fig. 2C): a unimodal region with an origin peak (denoted by U(1OP)) and a bimodal region with an origin peak as well as a non-origin peak (denoted by B(1OP+1NOP)). Additionally, the heatmap in Fig. 2C shows the difference between the peak and valley (DPV) in the bimodal distribution, whereas the value is defined as zero in the unimodal distribution. We observe that when both the forward transition rate $k_{f}$ and backward transition rate $k_{b}$ are substantially larger, the DPV of the bimodal distribution is also substantially larger (as indicated by the yellow region in region ii), implying a stronger effect of bimodality. Figure 2D illustrates 2 representative unimodal and bimodal distributions of initiation times. Comparing Fig. 2A and C, we observe that the distributions of nascent RNA can exhibit multiple modes within the bimodal region of initiation times. Similar conclusions were found when adjusting the transition rate $k_{f}$ and the initiation rate μ over a larger region of the parameter space (Fig. S2). The diversity of nascent RNA distribution for different initiation-time regions indicates that the initiation and elongation regulate transcription dynamics in a coordinated fashion.

Following a comprehensive analysis of the relationship between these 2 types of distributions, we proceed to investigate the potential of initiation-time data in facilitating model identification, with the results presented in Fig. 2E. We observe that 2 identical nascent RNA distributions arising from different model parameters exhibit completely different characteristics of initiation-time distribution: one is bimodal, while the other is unimodal, indicating that distributions of initiation times can help distinguish the underlying mechanisms of transcriptional regulation. This finding suggests that the use of nascent RNA data alone is insufficient for characterizing transcription dynamics. Instead, initiation-time data offer important insights into the regulation of gene expression. Fitting models to static gene expression data has been used to analyze dynamic behaviors, but this approach faces the challenge of model unidentifiability. Our results show that leveraging initiation-time data can effectively overcome the limitations of relying solely on expression data, thereby enhancing the accuracy of model identification.

### Incorporating initiation-time data can accurately infer the initiation rate and elongation time

The measurement of kinetic parameters represents a fundamental challenge in the study of gene expression. The parameters previously estimated using standardized methods based on static gene expression data are relative parameters that do not accurately reflect the true transcription dynamics [31,33,34]. A natural question arises: can adding initiation-time data help accurately estimate the transcriptional parameters? Furthermore, for data generated by either a 2-state model or other multistate models, is the inference of transcriptional parameters model independent when incorporating initiation-time data?

**Fig. 3.**
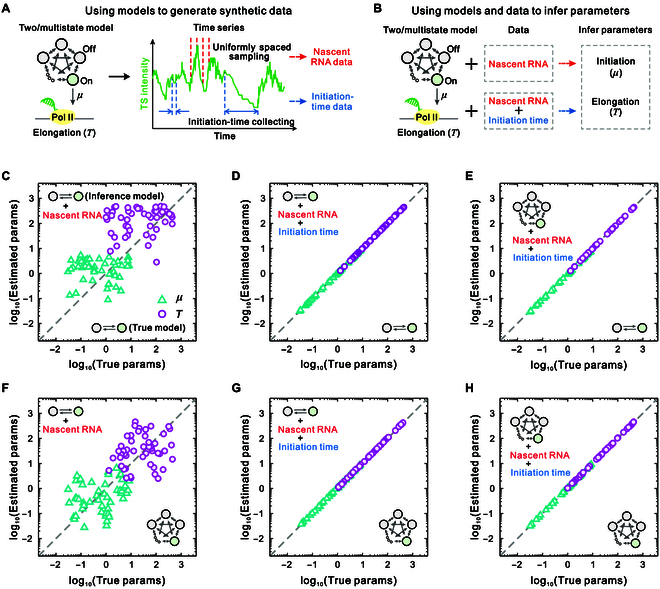
Parameter estimates using nascent RNA data (referred to as method I) and nascent RNA data combined with initiation-time data (referred to as method II) from 2-state and multistate models, with inference performed using these various models. (A) A schematic illustration of synthetic time series data generated using 2-state and multistate models, from which nascent RNA data and initiation-time data are collected. (B) Illustration of parameter estimations using 2-state and multistate models and 2 inference methods. (C) Parameters estimated using the 2-state model and method I vs. true parameters for datasets generated by the 2-state model. (D) Parameters estimated using the 2-state model and method II vs. true parameters for datasets generated by the 2-state model. (E) Parameters estimated using multistate models and method II vs. true parameters for datasets generated by the 2-state model. (F) Parameters estimated using the 2-state model and method I vs. true parameters for datasets generated by multistate models. (G) Parameters estimated using the 2-state model and method II vs. true parameters for datasets generated by multistate models. (H) Parameters estimated using multistate models and method II vs. true parameters for datasets generated by multistate models. In panels (C) to (H), the parameter sets are randomly generated from a large region of parameter space: $\lambda_{\mathrm{kl}}\in \text{Uniform}\left(0.01,10\right)$, $\mu \in \text{Uniform}\left(0.01,10\right)$, and $T\in \text{Uniform}\left(1,500\right)$. params, parameters.

To address these questions, we use 2-state and multistate (3-state to 10-state) models to generate time series data by the stochastic simulation algorithm (SSA) [69], from which we collect nascent RNA data using uniformly spaced sampling and initiation-time data by calculating the time intervals between consecutive initiation events (Fig. 3A). Then, we use different models to infer transcriptional parameters and perform a comparative benchmarking, where one approach uses only nascent RNA data for inference, while the other approach utilizes both nascent RNA data and initiation-time data for inference (Fig. 3B). It is noteworthy that the parameters estimated by these 2 methods are absolute rate parameters, without the imposition of any additional constraints.

To assess the accuracy of parameter inference, we compare the results obtained from different inference methods. Figure 3C to H show the inferred results for the 2 transcriptional parameters: the initiation rate μ and elongation time T, obtained from the 2 inference methods. First, Fig. 3C and F exhibit the estimated parameters using the 2-state model based on nascent RNA data generated by both 2-state and multistate models, respectively. From these figures, we observe that the estimations of these 2 parameters are quite poor, even when data generated by the 2-state model are used. The same conclusion is obtained when multistate models are employed for inference (Fig. S3). We then examine the effects of incorporating initiation-time data into the inference. Figure 3D, E, G, and H show the results of using different models for inference based on datasets generated by 2-state and multistate models. From these figures, we observe that, whether using the 2-state model or the multistate models for inference and regardless of the model from which the data originates, incorporating initiation-time data allows for accurate inference of the initiation rate and elongation time. These results suggest that the inference of these 2 transcriptional parameters is model independent when incorporating initiation-time data.

To further explore the effects of incorporating initiation-time data on the distributions of initiation times and nascent RNA counts, we plotted these 2 distributions using the estimated parameters (Fig. S4). The results suggest that incorporating initiation-time data is an effective method for accurately inferring the initiation rate and elongation time, regardless of whether the inferred distributions of initiation times and nascent RNA counts match the true distributions. In summary, incorporating initiation-time data can overcome the theoretical limitations of inferring transcriptional kinetic parameters from steady-state gene expression data.

### Testing theory and inference methods using synthetic imaging data

**Fig. 4.**
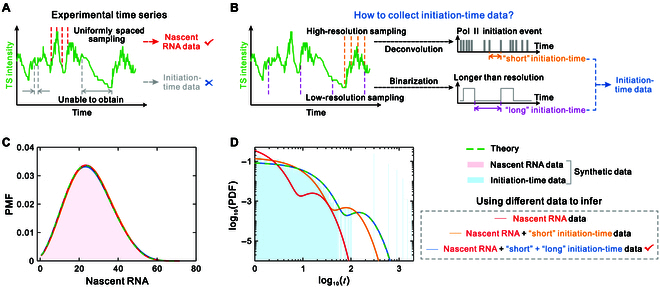
Testing the accuracy of theory and inference methods using synthetic short and long imaging data. (A) Collection of nascent RNA data by uniformly spaced sampling. (B) Collection of initiation-time data including “short” initiation times obtained through deconvolution and “long” initiation times processed by binarization. (C) The estimated nascent RNA distributions using 3 inference methods, the true nascent RNA distribution based on theory, and the histogram of nascent RNA distribution from uniformly spaced sampling data. (D) The estimated initiation-time distributions using 3 inference methods, the true initiation-time distribution based on theory, and the histogram of initiation-time distribution from collected short and long image data. The parameters used for the model produced data and the estimated parameters from 3 inferred methods are listed in Table S1. In addition, the parameters for the deconvolution algorithm are listed in Methods.

The applicability of the proposed theory and inference methods has been tested using simulated time series data, from which initiation times and nascent RNA counts were directly collected. A natural question that arises is whether these theory and inference methods can be applied to live-cell imaging data. For single-molecule imaging data, nascent RNA data can be acquired through uniform spatial sampling (Fig. 4A); however, initiation-time data cannot be directly obtained because multiple polymerases transcribe simultaneously at a given time for the same transcription site, and the signal from one polymerase does not appear immediately after initiation due to the involvement of multiple time scales [39]. To address this issue, we artificially generate imaging datasets that mimic real biological situations for testing, with the method for collecting the initiation-time data shown in Fig. 4B. Specifically, we first apply the deconvolution algorithm to short high-resolution movies and collect the “short” initiation-time data [38]. Then, we measure the long inactive periods below a threshold corresponding to 2 nascent RNAs over long low-resolution movies and collect the “long” initiation-time data (see Methods).

To verify the effectiveness of this method for imaging data analysis, we used a 5-state loop model to generate 500 trajectories based on the SSA, collecting nascent RNA data as well as “short” and “long” initiation-time data for inference. As a comparison, we employed 3 methods to infer model parameters: (I) using only nascent RNA data, (II) using nascent RNA data and short initiation-time data, and (III) using nascent RNA data as well as both short and long initiation-time data. Based on the inferred parameters, we plotted the estimated distributions of nascent RNA counts and initiation times and compared them with the actual distributions (Fig. 4C and D and Fig. S5). We observed that the estimated nascent RNA distributions fit the actual distributions well, regardless of the method used for inference (Fig. 4C and Fig. S5B and E). However, the estimated distributions of initiation times exhibited marked differences across the 3 inference methods: only the reconstructed initiation-time distributions using method III, i.e., nascent RNA data plus short and long initiation-time data, closely matched the true distributions, even though the histograms of initiation-time distributions obtained from the integration of short and long data differ substantially from the true distributions (Fig. 4D and Fig. S5C and F). Furthermore, we present the histograms of the initiation time distributions using unprocessed simulated data and find that the estimated distributions using integrated short and long imaging data fit the histograms well (Fig. S5A, D, and G). In summary, the results from synthetic imaging data suggest that our method provides an ideal framework for quantifying transcriptional dynamics in live cells.

### Analysis of live-cell HIV-1 experimental data indicates that Tat not only stimulates initiation but also promotes elongation

**Fig. 5.**
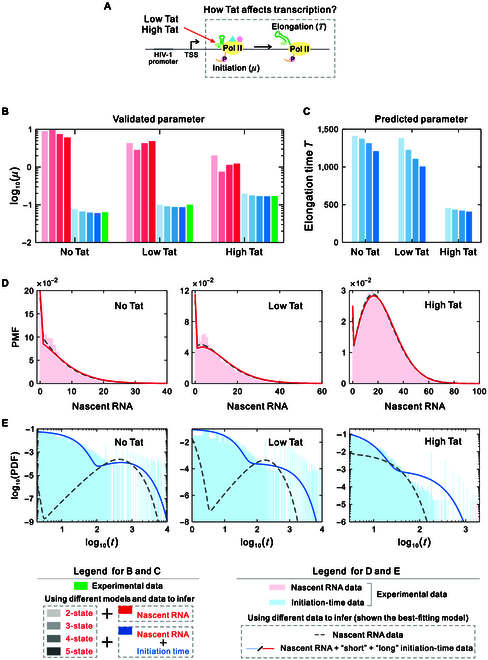
Results obtained by analyzing live-cell HIV-1 transcription data using 2-state and multistate models. (A) Diagram of Tat’s effect on HIV-1 transcription. (B) Estimates of initiation rates using only nascent RNA data (method I) and using both nascent RNA data and integrated short and long initiation-time data (method II) for 3 cell lines. (C) Predictions of elongation time using both nascent RNA data and integrated short and long initiation-time data for 3 cell lines. (D) The inferred distributions of nascent RNA from actual data using the best-fitting models from method I and method II for 3 cell lines. (E) The inferred distributions of initiation times from actual data using the best-fitting models from method I and method II for 3 cell lines. In addition, the estimated parameters from the 2 inferred methods for 3 cell lines are listed in Tables S2 and S3.

Here, we apply the proposed theory and inference methods to live-cell HIV-1 transcription data, which include 3 different datasets corresponding to high-Tat, low-Tat, and no-Tat cells [38]. The HIV-1 viral protein Tat is a pleiotropic factor that induces chromatin remodeling and recruits elongation-competent transcriptional complexes to the viral promoter, playing a crucial role in regulating both the initiation and elongation of HIV-1 expression [55,70]. Previous experiments have explored the effect of Tat on latency by measuring the initiation rates [38], yet the quantitative impact of Tat on elongation times remains unclear (Fig. 5A). Predicting elongation times could provide a deeper understanding of the replication capacity of HIV-1 under different conditions, which would be of considerable value for controlling the reactivation of latent viruses.

We use a 2-state model and multistate loop models, combining the initiation-time distribution and steady-state nascent RNA distribution to reproduce the live-cell transcription data. In our analysis, the nascent RNA data are obtained through uniform sampling of the movies, while the initiation-time data are collected by integrating short high-resolution sampling with long low-resolution sampling. Specifically, we deconvolve the short high-resolution movies with a temporal resolution of 3 s per frame and process the long low-resolution movies with a temporal resolution of 3 min per frame for these 3 types of cell lines (see Methods). The thresholds of signal intensity for separating active and inactive periods in the long movies are 500 for high Tat, 300 for low Tat, and 250 for no Tat. For each cell type, we performed inference using MLE to identify the optimized parameter sets (see Methods). We also performed inference using only nascent RNA data and compared the accuracy of these 2 inference methods with experimental results.

Next, we analyze the impact of incorporating initiation-time data into the estimation of HIV-1 transcription dynamics. Figure 5B shows estimated initiation rates without (red) and with (blue) the addition of initiation-time data for the 3 cell lines and compares these with the experimental results (green). We observe that the inference incorporating initiation-time data provides an accurate estimation of the initiation rates, whereas the inference using only nascent RNA data gives very poor estimates of the initiation rates, which can lead to differences by orders of magnitude. In particular, we found that, regardless of the model used for inference, incorporating initiation-time data can accurately estimate the initiation rates (comparing the bars from 2-state to 5-state models). Figure 5C shows the predicted elongation times for the 3 cell lines when initiation-time data are incorporated. We find that the elongation time in the high-Tat cells is substantially shorter than that observed in the low-Tat and no-Tat cells, implying that Tat plays a role in promoting the elongation of viral RNA transcription. Similarly, we found that the predictions of elongation time exhibit minimal discrepancy regardless of the model used for inference (comparing the bars from 2-state to 5-state models). The question of whether Tat stimulates initiation as well as elongation has been a topic of considerable debate [54,70,71]. Our inference incorporating initiation-time data indicates that Tat not only stimulates initiation but also promotes the elongation of viral transcription.

In addition, we find that the 2-state model provides the optimal fit for high-Tat and low-Tat cells, whereas the 3-state model is the optimal fit for no-Tat cells in the inference combining short and long initiation-time data (Table S2). These results suggest that HIV-1 transcription controls latency exit by altering the number of silent states of the promoter. However, the 2-state model is the optimal fit for the high-Tat cell lines, while the 5-state model is the optimal fit for both low-Tat and no-Tat cell lines in the inference using only nascent RNA data (Table S3). These findings suggest that inferences based solely on nascent RNA data may result in the inappropriate selection of models, leading to poor predictions. Figure 5D and E show the comparisons between the 2 inference results of nascent RNA distributions and initiation-time distributions obtained using the best-fitting models and the experimental data for 3 cell lines, respectively. We observe that both estimated distributions of nascent RNA fit the actual data well, whereas the estimated distributions of initiation times exhibit substantial differences (as compared to the solid and dashed curves). In addition, in the integrated inference of nascent RNA data and initiation-time data, we found that the initiation-time distributions estimated by the optimal models for the no-Tat and low-Tat cell lines are bimodal and span multiple time scales. In contrast, the initiation-time distribution estimated by the optimal model for high-Tat cell lines is unimodal and extends over a smaller time scale. These results indicate that Tat regulates HIV-1 transcription by modulating the stochastic timing of initiation. Our results obtained in Fig. 5 show that the simultaneous prediction of the initiation rate and elongation time may provide valuable insights into therapeutic strategies for latent infections of HIV-1 [8].

## Discussion

Previous studies using single-molecule imaging have shown that HIV-1 transcription occurs across a range of time scales [38,39], which is critical for the establishment, maintenance, and reactivation of latency. Therefore, new modeling and statistical inference methods for dealing with HIV-1 transcription distributed across multiple time scales are required. In this study, we first developed a general theoretical framework for quantifying HIV-1 transcription at the single-molecule level. We then introduced a multistate model that incorporates viral promoter, initiation, and elongation processes. Initiation determines the onset of transcription events, while elongation controls nascent RNA production, and both are key steps in HIV-1 transcription [2,8,54,72]. Our theoretical analysis and numerical simulations across a large parameter space showed that the distributions of initiation times can exhibit unimodality or bimodality, while the nascent RNA distributions can display unimodality, bimodality, or multimodality. Notably, the multimodal distributions emerge only in the bimodal region of the initiation-time distribution. These results suggest that initiation and elongation regulate gene expression in a coordinated manner. Furthermore, we found that stochastic models with completely different initiation-time distributions can exhibit the same steady-state distribution, suggesting that leveraging initiation-time data can enhance model identification.

To demonstrate the importance of incorporating initiation-time data for predicting gene regulatory dynamics, we propose a statistical inference method that integrates nascent RNA data and initiation-time data. We generated multiple datasets using different models across a broader parameter space. The results obtained from these synthetic datasets show that the 2-state model is able to accurately estimate the absolute initiation rate and elongation time when initiation-time data are incorporated. The 2-state model has been widely used to interpret single-cell expression data [22,28,63,73] and has achieved predictive understanding, especially for real-time observations [24,74–76]. Our results show that the incorporation of initiation-time data helps explain the prevalence of the 2-state model in gene expression studies, despite its inability to fully capture the complex dynamics of gene regulation [77–80]. Furthermore, we found that the initiation rate and elongation time can also be accurately estimated simultaneously using multistate models. These findings highlight the importance of incorporating initiation-time data in predicting gene regulatory dynamics.

Recent imaging techniques allow real-time observations in live cells, and the resulting imaging traces have provided temporal information about gene expression [81,82]. However, analytical frameworks for extracting valuable information from live imaging are still lacking. To test whether our method can be applied to single-molecule imaging analysis, we generated artificial imaging data to simulate real biological conditions. We collected initiation timing data by performing deconvolution analysis on short movies and measuring the inactive periods of long movies using a defined threshold. Our results show that our method still accurately inferred transcription dynamics, even though the histograms of the reconstructed initiation-time distributions from both short and long movies differ markedly from the true distributions (Fig. 4 and Fig. S5). At the same time, our results also indicate that using only short data may be insufficient, even if the time scales of the transcription processes are not considerably different.

Finally, our method was applied to HIV-1 transcriptional imaging data, from which the initiation-time data and nascent RNA data were extracted and collected. We performed computational analyses by integrating these 2 types of data with mathematical models and found that the viral factor Tat not only stimulates transcription initiation but also promotes transcription elongation, thus confirming the dual role of Tat in HIV-1 transcriptional regulation [54,55]. Furthermore, our inference revealed that the optimal model in no-Tat cells is a 3-state model, whereas in high-Tat cells it is a 2-state model, suggesting that Tat induces viral reactivation and latency exit by altering the number of silent states of the promoter. These insights provide a valuable connection to the latency and reactivation of HIV-1. Specifically, the initiation and elongation dynamics may play a key role in regulating the transition between latent and active infections. Viral latency is a major challenge for viral eradication in combined antiretroviral therapy [83,84]. Recently, 2 potential strategies for eliminating latent reservoirs have been proposed: the “block-and-lock” strategy, which aims to permanently silence all latent proviruses [85], and the “shock-and-kill” strategy, which focuses on the complete eradication of the viral reservoir [86]. By quantitatively analyzing HIV-1 transcription imaging data, our approach offers new insights into the mechanisms underlying these strategies and can help develop more precise therapeutic interventions. These interventions could enhance treatment effectiveness and ultimately address the major challenge of viral latency.

The simultaneous measurement of initiation and elongation rates is essential for a comprehensive understanding of transcriptional regulation. Methods that estimate kinetic parameters from steady-state distributions of gene products should be treated with caution due to the neglect of temporal information [22,33,34,87]. While these methods achieve excellent fits for steady-state distributions, they may make poor predictions for kinetic parameters. Recently, several studies have used initiation-time data to analyze transcription dynamics [35,36,38]. However, it is difficult to fully reveal the transcriptional regulatory mechanisms because downstream processes such as elongation are ignored. Our method, which integrates initiation-time data with nascent RNA data, can address the limitations of using only one type of data and can simultaneously predict initiation rate and elongation time, providing a comprehensive understanding of gene expression. Compared to static single-cell data, single-molecule imaging data may be relatively limited in both quantity and quality due to the constraints of live imaging technology and experimental challenges. The lack of adequate tools for analyzing limited data may lead to poor model fits and meaningless predictions [88]. Our theoretical and inferential methods, as well as our results, make it possible to extract meaningful predictions from limited imaging traces.

While the current study provides valuable insights, there are several aspects that could be further explored to refine our model and enhance its biological accuracy. First, in our stochastic model, we assume that elongation time is deterministic. However, elongation following RNA polymerase pausing, backtracking, and release can be complex, and elongation time can vary between and within genes [18,89,90]. It would be interesting to explore the effects of relaxing the assumption of deterministic elongation. Second, our stochastic model does not explicitly consider chromatin remodeling, RNA polymerase pausing, and other regulatory factors, but incorporated them into our multistate modeling framework. More detailed biological processes help characterize the multi-level regulatory interactions, which are critical for understanding HIV-1 latency and reactivation [72,91]. Third, we used a multistate model with a single active state to characterize HIV-1 transcription dynamics. It has been reported that multiple active states have been introduced into modeling to explain experimental phenomena [78,92]. In addition, a 2-state system where the times that the promoter dwells in both the on and off states are arbitrary random variables has also been proposed to analyze gene expression dynamics [93–95]. Exploring different modeling approaches helps enhance the robustness of the obtained results and deepen our understanding of both the model assumptions and the underlying biological mechanisms. Fourth, in data integration inference, we assume that initiation times and nascent RNA counts are independent. However, initiation events and gene product generation are often temporally coupled. Therefore, a more general joint likelihood function and an improved inference framework are needed to overcome potential biases in parameter estimation that might arise from the independence assumption.

In conclusion, integrating initiation-time data and nascent RNA data from single-molecule imaging can provide valuable information on gene expression dynamics. This approach could have broader applications in areas such as disease and development [1,96–98]. More detailed biological processes, such as pausing and termination [62,99], nuclear export [100,101], cell size effects [102,103], and protein translation [104,105], should be incorporated to explore the multi-level regulation of gene expression in future investigations.

## Methods

### Framework overview

This paper aims to develop novel methods that can simultaneously infer transcription initiation and elongation dynamics from single-molecule imaging data to explore the molecular mechanisms regulating HIV-1 transcription. The framework consists of 2 parts: a general stochastic model for characterizing HIV-1 transcription across multiple time scales and a data integration method for simultaneously inferring the initiation rate and elongation time. The general stochastic model integrates the processes of gene promoter activity, initiation, and elongation. The initiation-time distribution and nascent RNA distribution are obtained by solving the master equations. The data integration method combines initiation-time data and nascent RNA data, both of which can be collected from the same time series. When the proposed methods were applied to the imaging data from live cells, nascent RNA data were obtained through uniform sampling, while initiation-time data were collected from short movies using the deconvolution method and from long movies by binarizing the signal. Furthermore, a data integration inference algorithm based on MLE is proposed for estimating kinetic parameters from the joint distribution of these 2 types of data. Finally, the theory and method are tested with synthetic imaging data that simulate real biological conditions and are then applied to HIV-1 transcription data.

### Construction of a general stochastic model

To quantify the kinetic parameters from imaging data, a multistate model is introduced to characterize the HIV-1 transcription process (Fig. 1), where the gene promoter has multiple (N−1) inactive (off) states and one active (on) state. The corresponding biochemical reactions are listed in the Table. It should be noted that if the transitions between inactive states as well as active and inactive states are sequential and irreversible, the multistate model becomes a loop model [78]. In particular, if the gene promoter has only an off state, the multistate model reduces to the classical 2-state model [29]. Therefore, the multistate model introduced here includes the transcription model that was previously studied. Compared to the simple 2-state or 3-state models, the multistate model can recapitulate a wide range of HIV-1 promoter states and transcription dynamics.

**Table.** Biochemical reactions for a multistate model of HIV-1 transcription

Reactions	Description
$I_{k}{⇄}_{\lambda_{\mathrm{lk}}}^{\lambda_{\mathrm{kl}}}I_{l},\;k,l=1,2,\cdots ,N-1$	Transitions between inactive states
$I_{k}{⇄}_{\lambda_{\mathrm{Nk}}}^{\lambda_{\mathrm{kN}}}A,\;k=1,2,\cdots ,N-1$	Transitions between inactive and active states
$A\overset{\mu }{\to }A+\text{nascent}\;\mathrm{RNA}$	Transcription initiation
$\text{Nascent}\;\mathrm{RNA}\overset{T}{\Rightarrow }\Phi$	Transcription elongation

### Computation of the initiation-time distribution

To analyze HIV-1 transcription initiation dynamics, the initiation-time distribution needs to be solved. By solving the chemical master equation that describes the evolution of the probability of the promoter states (refer to Eq. 1), the initiation-time distribution can be obtained as follows:

$$\begin{array}{c} f_{\mathrm{ini}}\left(t\right)=\sum_{j=1}^{n_{1}}\sum_{k=1}^{p_{j}}c_{\mathrm{jk}}\frac{t^{k-1}}{\left(k-1\right)!}e^{-\lambda_{j}t}+\\ \sum_{j=1}^{n_{2}}\sum_{k=1}^{q_{j}}d_{\mathrm{jk}}\frac{t^{k-1}}{\left(k-1\right)!}e^{-\eta_{j}t}\text{cos}\left(\sigma_{j}t\right)+\sum_{j=1}^{n_{2}}\sum_{k=1}^{q_{j}}e_{\mathrm{jk}}\frac{t^{k-1}}{\left(k-1\right)!}e^{-\eta_{j}t}\text{sin}\left(\sigma_{j}t\right) \end{array}$$ (5)

where all parameters involved are real constants and are described in Text S1.1. The analytical expressions of initiation-time distribution for several representative models are given in Text S1.2. Additionally, the initiation-time distribution of the multistate loop model can be obtained from Eq. 5 by setting $p_{j}=q_{j}=1$. Recently, several studies have explored the initiation-time distributions for several simple stochastic models [35,36,38,106], all of which are special cases of Eq. 5. Compared to the multi-exponential survival function approach (where the initiation-time distribution also follows a multi-exponential form) [38], our initiation-time distribution can capture complex transcription initiation dynamics across multiple time scales. In particular, the cosine and sine terms in Eq. 5 suggest that the initiation-time distribution can exhibit potential oscillatory behaviors, implying that the promoter is not simply switching between the on and off states but rather undergoing multiple intermediate states.

### Computation of the steady-state nascent RNA distribution

To analyze how transcription initiation and elongation coordinate gene expression, the steady-state nascent RNA distribution for the multistate model needs to be solved. By applying renewal theory and assuming a deterministic elongation time (refer to Eq. 3), the steady-state nascent RNA distribution can be derived as follows:

$$\begin{array}{c} \begin{array}{c} P\left(M=m\right)=\left\{\begin{array}{cc} \sum_{j=1}^{n_{1}}\sum_{k=1}^{p_{j}}C_{j,k,m}\frac{T^{k-1}}{\left(k-1\right)!}e^{-\lambda_{j}T}+\sum_{j=1}^{n_{2}}\sum_{k=1}^{q_{j}}\frac{T^{k-1}}{\left(k-1\right)!}e^{-\eta_{j}T}\left(D_{j,k,m}\text{cos}\left(\sigma_{j}T\right)+E_{j,k,m}\text{sin}\left(\sigma_{j}T\right)\right), & m=0,\\ \sum_{j=1}^{n_{1}}\sum_{k=0}^{p_{j}\left(m+1\right)-1}\frac{C_{j,k,m}T^{p_{j}\left(m+1\right)-1-k}e^{-\lambda_{j}T}}{\left(p_{j}\left(m+1\right)-1-k\right)!}+\sum_{j=1}^{n_{2}}\sum_{k=0}^{q_{j}\left(m+1\right)-1}\frac{T^{q_{j}\left(m+1\right)-1-k}e^{-\eta_{j}T}}{\left(q_{j}\left(m+1\right)-1-k\right)!}\left(D_{j,k,m}\text{cos}\left(\sigma_{j}T\right)+E_{j,k,m}\text{sin}\left(\sigma_{j}T\right)\right), & m\geq 1, \end{array}\right. \end{array} \end{array}$$ (6)

where $C_{j,k,m}$, and $D_{j,k,m}$ $E_{j,k,m}$ are real constants and are computed in Text S2. In particular, the steady-state nascent RNA distribution for the multistate loop model can be obtained from Eq. 6 by setting $p_{j}=q_{j}=1$. Previous studies have derived the analytical solutions for the distribution of nascent RNA for several simple stochastic models [63,106], all of which are special cases of Eq. 6. The 2 analytical results presented in Eqs. 5 and 6 provide a theoretical foundation for a comprehensive exploration of HIV-1 transcription dynamics.

### Computation of the steady-state binomial moments of nascent RNA distribution

The binomial moments $B_{k}$ of the steady-state nascent RNA distribution $P\left(M=m\right)$ are useful indicators for quantifying transcription dynamics. By introducing the probability-generating function, the steady-state binomial moments can be derived as follows:

Bk=∑j=1k+1Fj,kj−1!Tj−1+∑j=1n1Δ∑h=0pjΔk−1−1Gj,h,kTpjΔk−1−1−he−λjΔTpjΔk−1−1−h!+∑j=1n2Δ∑h=0qjΔk−1−1TqjΔk−1−1−he−ηjΔTqjΔk−1−1−h!Hj,h,kcosσjΔT+Kj,h,ksinσjΔT, (7)

where all parameters involved are real constants and are described in detail in Text S3. Furthermore, the central moments can be calculated from the binomial moments (Eq. S92 in Text S3). Therefore, the bimodal coefficient $\mathrm{BC}=1/\left(K-S^{2}\right)$, where $S=B_{3}^{c}/{\left(B_{2}^{c}\right)}^{3/2}$ is the skewness of nascent RNA, $K=B_{4}^{c}/{\left(B_{2}^{c}\right)}^{2}$ is the kurtosis, and $B_{k}^{c}$ represents the kth central moments, can be obtained from the steady-state moments.

### Collection of initiation-time data and nascent RNA data from synthetic data

To explore whether incorporating initiation-time data can accurately infer the initiation rate and elongation time, the method is tested using synthetic data. Parameter sets are generated from a large region of parameter space

$$\begin{array}{c} \begin{array}{c} \begin{array}{c} \left(\lambda_{\mathrm{kl}},\;\mu ,\;T\right)\in \left[\text{Uniform}\left(0.01,10\right),\;\text{Uniform}\left(0.01,10\right),\;\text{Uniform}\left(1,500\right)\right] \end{array} \end{array} \end{array}$$ (8)

Once a set of parameters is chosen, the SSA [69] is used to simulate the biochemical reactions of the multistate (2-state to 10-state) models in Table and generate 500 independent trajectories over a time period of 10^4^ s. From the same time series data, the initiation-time data are collected by recording the time intervals between successive initiation events, while the nascent RNA data are collected by uniform sampling.

### Data integration algorithm for estimating kinetic parameters

To estimate kinetic parameters using both initiation-time data and nascent RNA data, a data integration method based on MLE is proposed, which utilizes the joint distribution of initiation times and nascent RNA counts predicted by the multistate model. Let $P\left(\tau ,\;m;\;\theta \right)$ be the joint distribution of 2 types of data, where $\theta =\left(\lambda_{\mathrm{kl}},\;\mu ,\;T\right)$ is the parameter vector of the multistate model to be estimated. For convenience, the search region for optimal parameters is restricted to be the same as in Eq. 8. The total likelihood function is defined as $L\left(\theta \right)=\prod_{i,j}P\left(\tau_{i},\;m_{j};\;\theta \right)$ with $\tau_{i}$ being the ith initiation data and $m_{j}$ being the jth nascent RNA data. The inference can be formulated as the following optimization problem:

$$\begin{array}{c} \underset{\theta }{\text{arg}}\text{min}\left(-L\left(\theta \right)\right)=\underset{\theta }{\text{arg}}\text{min}\left(-\sum_{i,j}\text{ln}P\left(\tau_{i},\;m_{j};\;\theta \right)\right). \end{array}$$ (9)

Suppose that the occurrence of transcription initiation events is independent of the elongation events in this study, so it is reasonable to assume that joint distribution $P\left(\tau_{i},\;m_{j};\;\theta \right)=f_{\mathrm{ini}}\left(\tau_{i};\;\theta \right)P\left(m_{j},\;\theta \right)$, with $f_{\mathrm{ini}}\left(\tau_{i};\theta \right)$ and $P\left(m_{j},\;\theta \right)$ being the marginal distribution of initiation times and nascent RNA counts, respectively. Then, the optimization problem becomes

$$\begin{array}{c} \underset{\theta }{\text{arg}}\text{min}\left(-L\left(\theta \right)\right)=\\ \underset{\theta }{\text{arg}}\text{min}\left(-\left(\sum_{i}\text{ln}f_{\mathrm{ini}}\left(\tau_{i};\;\theta \right)+\sum_{j}\text{ln}P\left(m_{j};\;\theta \right)\right)\right) \end{array}$$ (10)

where $f_{\mathrm{ini}}\left(\tau_{i};\;\theta \right)$ and $P\left(m_{j},\;\theta \right)$ are given by Eqs. 5 and 6, respectively. Here, the independent assumption of initiation times and nascent RNA counts decomposes the complex biological process into separately modeled components, making the problem more tractable.

To enable fast calculations for solving the optimization problem given a set of initial values and parameter intervals, finite state projection is used to solve the multistate model, and an efficient gradient-free optimization algorithm is employed to find the optimal parameters by maximizing the likelihood [107]. For each inference, a large iteration step $N_{\max}\geq 5,000$ is chosen as the termination condition for the optimizer. For comparison, the kinetic parameters are inferred only using the nascent RNA data, as many previous studies have done [33,87]. Consequently, the optimization problem becomes solely related to the steady-state distribution of nascent RNA, i.e., $\begin{array}{c} {\text{arg}}_{\theta }\text{min}\left(-L\left(\theta \right)\right)={\text{arg}}_{\theta }\text{min}\left(-\sum_{j}\text{ln}P\left(m_{j};\;\theta \right)\right) \end{array}$.

### Live-cell imaging data acquisition and processing

Three different live-cell imaging data of HIV-1 transcription are used in this study [38]. These 3 cell lines that express different levels of Tat all contained the 128xMS2 reporter integrated at the same chromosomal location. By monitoring the brightness of the transcription site over time, 2 types of movies were recorded to cover the entire temporal range of HIV-1 transcriptional fluctuations: short movies capture an image stack every 3 s for 15 to 20 min, while long movies last for 8 h with a rate of one image stack every 3 min.

### Collection of initiation-time data and nascent RNA data from live-cell imaging data

The single transcription initiation event cannot be directly detected in the live-cell imaging data, because multiple polymerases transcribe simultaneously at a given time for the same transcription site. In addition, HIV-1 transcription involves multiple time scales, and the signal from one polymerase does not appear immediately after initiation. Therefore, initiation-time data need to be collected from both short movies and long movies. For the short movie, a deconvolution method is applied to reconstruct individual transcription initiation events, in which polymerase positions are determined using a genetic algorithm with a local optimization procedure [38] (see Text S5 for details). In the algorithm, the polymerase dwell time on the DNA is calculated using the single polymerase pattern according to $\left(l_{\mathrm{pre}}+l_{\mathrm{seq}}+l_{\text{post}}\right)/V_{\mathrm{pol}}+t_{\mathrm{pol}}$, where $l_{\mathrm{pre}}$, $l_{\mathrm{seq}}$, and $l_{\text{post}}$ represent the length in base pairs of the 3 sequences: before the MS2 sequence, the MS2 sequence, and the sequence after MS2, respectively; $V_{\mathrm{pol}}$ and $t_{\mathrm{pol}}$ are the elongation speed of the polymerase and polyadenylation time, respectively. The parameters are taken as $l_{\mathrm{pre}}=700\;\mathrm{bp}$, $l_{\mathrm{seq}}=5,800\;\mathrm{bp}$, $l_{\text{post}}=1,600\;\mathrm{bp}$, $V_{\mathrm{pol}}=67\;\mathrm{bp}/s$, and $t_{\mathrm{pol}}=100\;s$. Consequently, the “short” initiation-time data are collected from reconstructed transcription initiation events. For the long movie, the signal is binarized and an intensity threshold is defined to identify the active and inactive periods, with the “long” initiation-time data collected by counting the inactive periods below the threshold. Finally, the distribution of initiation times can be reconstructed from the collected short and long initiation-time data. In addition, nascent RNA data are collected through uniform sampling from imaging data.

### Kinetic parameter identifiability

The method proposed in this study provides a theoretical guarantee for the inference of kinetic parameters. The model parameters can be identified by the coefficients of the Laplace transform ${\tilde{f}}_{\mathrm{ini}}\left(s\right)$ (refer to Eq. S2 in Text S1) and the steady-state binomial moments $B_{k}$ of nascent RNA distribution in Eq. 7. Specifically, assume that there are l kinetic parameters $\theta_{1},\theta_{2},\cdots ,\theta_{l},$ plus the elongation time T to be estimated, and then we can establish the following relation:

$$\begin{array}{c} H\left(\theta_{1},\;\theta_{2},\;\cdots ,\;\theta_{l},\;T\right)=\\ G\left(a_{1},\;a_{2},\;\cdots ,\;a_{N};\;b_{1},\;b_{2},\;\cdots ,\;b_{N};\;B_{1},\;B_{2},\;\cdots B_{k}\right) \end{array}$$ (11)

where $a_{i},b_{i}$, i=1,2,⋯,N, are the coefficients of the rational function ${\tilde{f}}_{\mathrm{ini}}\left(s\right).$ H and G are $\left(2N-1+k\right)$-dimensional column vectors. By simple calculations, the $\left(l+1\right)$ model parameters can be identified from Eq. 11. Observe that if l≤2N−1, the kinetic parameters $\theta_{1},\theta_{2},\cdots ,\theta_{l}$ can be determined only by the coefficients $a_{1},a_{2},\cdots ,a_{N}$ and $b_{1},b_{2},\cdots ,b_{N}$; furthermore, we need the first binomial moment $B_{1}$ to determine the elongation time T. If l>2N−1, we would need more binomial moments $B_{1},B_{2},\cdots ,B_{l+2-2N}$ to identify the model parameters. As the calculation of the high-order moments of the steady-state distribution is very complicated, the proposed method reduces the difficulty of identifying model parameters compared to the inference method that uses only nascent RNA data. In addition, the kinetic parameters for several representative models, such as the on–off model and the multistate loop models, are identified in Text S4. In particular, our method provides an explanation for the interchangeable rate constants of promoter state transitions inferred from static gene expression data (see Text S4 for details).

## Data Availability

The 3 live-cell imaging data of HIV-1 transcription are available from Tantale et al. [38]. Data generated and analyzed during the current study are available from the corresponding author upon reasonable request.

## Supplementary Materials

Supplementary 1 Texts S1 to S5 Figs. S1 to S5 Tables S1 to S3
