# Supplementary material for: Deciphering HIV-1 Transcription Initiation and Elongation from Single-Molecule Imaging Data
Source: Research (Wash D C). 2025 Mar 31;8:0645. doi: 10.34133/research.0645 (PMC12692740; doi:10.34133/research.0645)
Supplement: Supplementary 1 — Texts S1 to S5 Figs. S1 to S5 Tables S1 to S3 [file research.0645.f1.pdf]

Supplementary Material

Deciphering HIV-1 Transcription Initiation and Elongation from Single-Molecule Imaging Data

Xiyan Yang[1,†] , Zihao Wang[2,3,†], Changhong Shi[4] , Tianshou Zhou[2,3] , and Jiajun Zhang[2,3,]

[1] School of Financial Mathematics and Statistics, Guangdong University of Finance, Guangzhou 510521, P. R. China

[2] Guangdong Province Key Laboratory of Computational Science, Guangzhou 510275, P. R. China

[3] School of Mathematics, Sun Yat-sen University, Guangzhou 510275, P. R. China

[4] The State Key Laboratory of Respiratory Disease, School of Public Health, Guangzhou Medical University, Guangzhou 510182, P. R. China

†These authors contributed equally to this work.

[*] To whom correspondence should be addressed.

Email: zhjiajun@mail.sysu.edu.cn

Content

Supplementary Text 3

Section S1. Deriving the initiation time distribution 3

S1.1 Deriving the initiation time distribution for general initiation mechanisms 3

S1.2 Deriving the initiation time distribution for several representative initiation mechanisms 5

Section S2. Deriving the steady-state distribution of nascent RNA 10

Section S3. Deriving the binomial moments of steady-state nascent RNA distribution 15

Section S4. Identifying the kinetic parameters for several representative models 18

S4.1 Identifying the kinetic parameters for the ON-OFF model 18

S4.2 Identifying the kinetic parameters for the multi-state loop models 18

Section S5. Description of deconvolution algorithm 20

Supplementary Figures 22

Figure S1 Comparison of steady-state nascent RNA distributions using analytical and numerical solutions. .. 22

Figure S2 Stochastic bifurcation diagrams for nascent RNA distribution and initiation distribution 23

Figure S3 Parameter estimates using nascent RNA data from two-state and multi-state models 24

Figure S4 Comparison of the estimated distributions with the actual distributions for synthetic data. 24

Figure S5 Testing the accuracy of theory and inference methods using synthetic imaging data. 25

Supplementary Tables 26

Table S1 Comparison of parameters used in the models and those estimated from three inference methods ... 26

Table S2 Parameters inferred from the integration of nascent RNA and initiation time data for three cell lines27

Table S3 Parameters inferred from nascent RNA data for three cell lines 28

Supplementary References 29

Supplementary Text

Section S1: Deriving the initiation time distribution

In this section, we derive the initiation time distribution, which includes general initiation mechanisms and several representative initiation mechanisms.

S1.1: Deriving the initiation time distribution for general initiation mechanisms

In the following, we derive the analytical expression of initiation time distribution for general initiation mechanisms. Taking the Laplace transform of initiation time distribution in equation (2) in the main text, which yields

$$\tilde{f}_{\text{ini}}(s) = \mathbf{u}_{\text{ini}}\left(s\mathbf{I} - \mathbf{A}_{\text{ini}}\right)^{-1}\mathbf{P}(0). \tag{S1}$$

Substituting the initial value $\mathbf{P}(0) = (0,0,\cdots,0,1)$ into Eq. (S1), $\tilde{f}_{\text{ini}}(s)$ can further be written as the following rational function

$$\tilde{f}_{\text{ini}}(s) = \frac{a(s)}{b(s)} = \frac{a_1 s^{N-1} + a_2 s^{N-2} + \cdots a_{N-1}s + a_N}{s^N + b_1 s^{N-1} + b_2 s^{N-2} + \cdots b_{N-1}s + b_N}, \tag{S2}$$

where $a_1,\cdots,a_N,b_1,\cdots,b_N$ are real constants, and $a_N = b_N$ according to the conservative condition. Note that the coefficients in the denominator $b(s)$ are real, we write the Laplace transform in the form

$$\tilde{f}_{\text{ini}}(s) = \frac{a_1 s^{N-1} + a_2 s^{N-2} + \cdots a_{N-1}s + a_N}{\left(s+\lambda_1\right)^{p_1}\cdots\left(s+\lambda_{n_1}\right)^{p_{n_1}}\left(\left(s+\eta_1\right)^2+\sigma_1^2\right)^{q_1}\cdots\left(\left(s+\eta_{n_2}\right)^2+\sigma_{n_2}^2\right)^{q_{n_2}}}, \tag{S3}$$

where $-\lambda_1,\cdots,-\lambda_{n_1}$ denote possible real poles of $b(s)$ with multiplicity $p_1,\cdots,p_{n_1}$, and $-\eta_1 \pm \sigma_1\mathrm{i},\cdots,-\eta_{n_2} \pm \sigma_{n_2}\mathrm{i}$ are possible complex poles of $b(s)$ with multiplicity $q_1,\cdots,q_{n_2}$. The partial fraction decomposition of $\tilde{f}_{\text{ini}}(s)$ has the following form

$$\tilde{f}_{\text{ini}}(s) = \sum_{j=1}^{n_1}\sum_{k=1}^{p_j}\frac{c_{jk}}{\left(s+\lambda_j\right)^k} + \sum_{j=1}^{n_2}\sum_{k=1}^{q_j}\frac{d_{jk}+\mathrm{i}e_{jk}}{2\left(s+\eta_j+\mathrm{i}\sigma_j\right)^k} + \sum_{j=1}^{n_2}\sum_{k=1}^{q_j}\frac{d_{jk}-\mathrm{i}e_{jk}}{2\left(s+\eta_j\text{-}\mathrm{i}\sigma_j\right)^k}, \tag{S4}$$

where

$$\begin{aligned}
c_{jk} &= \frac{1}{\left(p_j-k\right)!}\frac{d^{\left(p_j-k\right)}}{ds^{\left(p_j-k\right)}}\left[\tilde{f}_{\text{ini}}(s)\left(s+\lambda_j\right)^{p_j}\right]\Bigg|_{s=-\lambda_j}, \\
d_{jk} &= \frac{1}{\left(q_j-k\right)!}\left\{\frac{d^{\left(q_j-k\right)}}{ds^{\left(q_j-k\right)}}\left[\tilde{f}_{\text{ini}}(s)\left(s+\eta_j+\mathrm{i}\sigma_j\right)^{q_j}\right]\Bigg|_{s=-\left(\eta_j+\mathrm{i}\sigma_j\right)} + \frac{d^{\left(q_j-k\right)}}{ds^{\left(q_j-k\right)}}\left[\tilde{f}_{\text{ini}}(s)\left(s+\eta_j-\mathrm{i}\sigma_j\right)^{q_j}\right]\Bigg|_{s=-\left(\eta_j-\mathrm{i}\sigma_j\right)}\right\}, \\
e_{jk} &= -\frac{1}{\left(q_j-k\right)!}\mathrm{i}\left\{\frac{d^{\left(q_j-k\right)}}{ds^{\left(q_j-k\right)}}\left[\tilde{f}_{\text{ini}}(s)\left(s+\eta_j+\mathrm{i}\sigma_j\right)^{q_j}\right]\Bigg|_{s=-\left(\eta_j+\mathrm{i}\sigma_j\right)} - \frac{d^{\left(q_j-k\right)}}{ds^{\left(q_j-k\right)}}\left[\tilde{f}_{\text{ini}}(s)\left(s+\eta_j-\mathrm{i}\sigma_j\right)^{q_j}\right]\Bigg|_{s=-\left(\eta_j-\mathrm{i}\sigma_j\right)}\right\}.
\end{aligned} \tag{S5}$$

To find these coefficients, we denote the possible real or complex pole of $b(s)$ in Eq. (S3) as $-x$ with multiplicity θ, more precisely, $x=\lambda_j, \theta=p_j$ for the real pole and $x=\eta_j \pm \mathrm{i}\sigma_j, \theta=q_j$ for the complex pole. In addition, the remain possible poles without considering multiplicities are denoted as $-x_1,-x_2,\cdots,-x_{N-\theta}$. Similarly, the possible poles of $a(s)$ in Eq. (S3) without considering multiplicities are denoted as $-y_1,-y_2,\cdots,-y_{N-1}$. Set

$$u_l(s)=\begin{cases}(s+y_l)/(s+x_l), & l=1,\cdots,N-\theta \\ s+y_l, & l=N-\theta+1,\cdots,N-1\end{cases}. \tag{S6}$$

Next, we define an auxiliary function $u(s)=\tilde{f}_{\mathrm{ini}}(s)(s+x)^{\theta}$, and substitute the above expression into $u(s)$, we have

$$u(s)=\prod_{l=1}^{N-1}u_l(s). \tag{S7}$$

If we denote the $n-$order derivative of $u(s)$ as $d^n u(s)/ds^n=(u(s))^{(n)}=\left(\prod_{l=1}^{N-1}u_l(s)\right)^{(n)}$, then we have

$$(u(s))^{(n)}=n!\sum_{r_1+\cdots r_{N-1}=n}\frac{(u_1(s))^{(r_1)}}{r_1!}\frac{(u_2(s))^{(r_2)}}{r_2!}\cdots\frac{(u_{N-1}(s))^{(r_{N-1})}}{r_{N-1}!}, \tag{S8}$$

with

$$(u_l(s))^{(r)}=\begin{cases}(-1)_r\dfrac{s+y_l}{(s+x_l)^{1+r}}\,{}_2F_1\left(-r,-1;-r;\dfrac{s+x_l}{s+y_l}\right), & l=1,2,\cdots,N-\theta \\ 1, & l=N-\theta+1,\cdots,N-1; r=1 \\ 0, & l=N-\theta+1,\cdots,N-1; r=2,3,\cdots,n\end{cases} \tag{S9}$$

where $(j)_r=j(j-1)\cdots(j-r+1)$ is the fall factorial, and ${}_2F_1(\bullet)$ is the Gaussian hypergeometric function. Substituting Eqs. (S8)-(S9) and $s=-x$ into Eq. (S5), we find these coefficients c_{jk}, d_{jk}, and e_{jk}.

To obtain the inverse Laplace transform of $\tilde{f}_{\mathrm{ini}}(s)$, we rewrite Eq. (S4) as follows

$$\begin{aligned}\tilde{f}_{\mathrm{ini}}(s)=&\sum_{j=1}^{n_1}\sum_{k=1}^{p_j}\frac{c_{jk}}{(s+\lambda_j)^k}+\sum_{j=1}^{n_2}\sum_{k=1}^{q_j}d_{jk}\left(\frac{1}{2(s+\eta_j+\mathrm{i}\sigma_j)^k}+\frac{1}{2(s+\eta_j-\mathrm{i}\sigma_j)^k}\right)\\ &+\sum_{j=1}^{n_2}\sum_{k=1}^{q_j}e_{jk}\mathrm{i}\left(\frac{1}{2(s+\eta_j+\mathrm{i}\sigma_j)^k}-\frac{1}{2(s+\eta_j-\mathrm{i}\sigma_j)^k}\right).\end{aligned} \tag{S10}$$

Taking the inverse Laplace transform of $\tilde{f}_{\mathrm{ini}}(s)$, and using the following identities

$$L^{-1}\left\{\frac{1}{\left(s+\lambda_j\right)^k}\right\}(t)=\frac{t^{k-1}}{(k-1)!}e^{-\lambda_j t},$$

$$L^{-1}\left\{\frac{1}{2\left(s+\eta_j+\mathrm{i}\sigma_j\right)^k}+\frac{1}{2\left(s+\eta_j-\mathrm{i}\sigma_j\right)^k}\right\}(t)=\frac{t^{k-1}}{(k-1)!}e^{-\eta_j t}\cos\left(\sigma_j t\right), \tag{S11}$$

$$L^{-1}\left\{\mathrm{i}\left(\frac{1}{2\left(s+\eta_j+\mathrm{i}\sigma_j\right)^k}-\frac{1}{2\left(s+\eta_j-\mathrm{i}\sigma_j\right)^k}\right)\right\}(t)=\frac{t^{k-1}}{(k-1)!}e^{-\eta_j t}\sin\left(\sigma_j t\right),$$

we get the initiation time distribution as follows

$$f_{\mathrm{ini}}(t)=\sum_{j=1}^{n_1}\sum_{k=1}^{p_j}c_{jk}\frac{t^{k-1}}{(k-1)!}e^{-\lambda_j t}+\sum_{j=1}^{n_2}\sum_{k=1}^{q_j}d_{jk}\frac{t^{k-1}}{(k-1)!}e^{-\eta_j t}\cos\left(\sigma_j t\right)+\sum_{j=1}^{n_2}\sum_{k=1}^{q_j}e_{jk}\frac{t^{k-1}}{(k-1)!}e^{-\eta_j t}\sin\left(\sigma_j t\right). \tag{S12}$$

Eq. (S12) implies that the distribution of initiation times in the multi-state model may be very complex and may involve multiple timescales.

S1.2: Deriving the initiation time distribution for several representative initiation mechanisms

A. The distribution of initiation times for the ON-OFF model

For the ON-OFF model, the promoter switches between an active (ON) state and an inactive (OFF) state. In this model, we assume that the transition rate from the OFF state to the ON state is k_{on}, while the transition rate from the ON state to the OFF state is k_{off}. The Laplace transform of the initiation time distribution has the following form

$$\tilde{f}_{\mathrm{ini}}(s)=\frac{a_1 s+a_2}{s^2+b_1 s+b_2}, \tag{S13}$$

with $a_1=\mu,\ b_1=k_{\mathrm{on}}+k_{\mathrm{off}}+\mu,\quad a_2=b_2=k_{\mathrm{on}}\mu.$ It is easy to get the roots of the characteristic equation $s^2+b_1 s+b_2=0$ as follows

$$-\lambda_1=\frac{-\left(k_{\mathrm{on}}+k_{\mathrm{off}}+\mu\right)-\sqrt{\left(k_{\mathrm{on}}+k_{\mathrm{off}}+\mu\right)^2-4k_{\mathrm{on}}\mu}}{2},-\lambda_2=\frac{-\left(k_{\mathrm{on}}+k_{\mathrm{off}}+\mu\right)+\sqrt{\left(k_{\mathrm{on}}+k_{\mathrm{off}}+\mu\right)^2-4k_{\mathrm{on}}\mu}}{2}. \tag{S14}$$

By simple computation, the distribution of initiation time has the following form

$$f_{\mathrm{ini}}(t)=c_1e^{-\lambda_1 t}+c_2e^{-\lambda_2 t}, \tag{S15}$$

with the coefficients

$$c_1=\frac{\lambda_1\left(\mu-\lambda_2\right)}{\lambda_1-\lambda_2},\quad c_2=\frac{\lambda_2\left(\lambda_1-\mu\right)}{\lambda_1-\lambda_2}. \tag{S16}$$

In addition, we can prove that $c_1>0,$ and $c_2>0.$ Note that $\lambda_1>\lambda_2,$ so it is sufficient to prove that

$$\lambda_2<\mu<\lambda_1. \tag{S17}$$

Combining Eq. (S14) and Eq. (S17), it is equivalent to proving that

$$\sqrt{(k_{\text{on}}+k_{\text{off}}+\mu)^2-4k_{\text{on}}\mu} > \mu-(k_{\text{on}}+k_{\text{off}}), \qquad (k_{\text{on}}+k_{\text{off}})-\mu < \sqrt{(k_{\text{on}}+k_{\text{off}}+\mu)^2-4k_{\text{on}}\mu}. \tag{S18}$$

It is easy to show that Eq. (S18) holds because $k_{\text{off}}>0, \mu>0$. Thus, we can obtain an important result: the distribution of initiation times in the ON-OFF model is always monotonically decreasing, which is very useful for further analyzing the steady-state distribution of nascent RNA.

B. The distribution of initiation times for an arbitrary three-state model

For a three-state model with arbitrary promoter switching, the Laplace transform of the initiation time distribution takes the following form

$$\tilde{f}_{\text{ini}}(s)=\frac{a_1 s^2+a_2 s+a_3}{s^3+b_1 s^2+b_2 s+b_3}. \tag{S19}$$

To find the explicit expression of initiation time distribution $f_{\text{ini}}(t)$, we first give the classification of roots of the following characteristic equation [1]

$$s^3+b_1 s^2+b_2 s+b_3=0. \tag{S20}$$

Set $D_2=b_1^2-3b_2$, $D_3=18b_1b_2b_3+b_1^2b_2^2-4b_1^3b_3-4b_2^3-27b_3^2$, we can show the numbers of real and imaginary roots of Eq. (S20) as follows

(1) $D_2>0\wedge D_3>0$, there are three district real roots:

$$-\lambda_j=2\sqrt[3]{r}\cos\frac{\varphi+2(j-1)\pi}{3}-\frac{1}{3}b_1, \qquad j=1,2,3 \tag{S21}$$

with $r=\sqrt{(b_1^2-3b_2)^3}\big/27$, $\varphi=\arccos\left[\left(27b_1b_2-6b_1^3-81b_3\right)\big/162r\right]$.

(2) $D_2>0\wedge D_3=0$, there are three real roots with a simple root and two repeated roots:

$$-\lambda_1=\frac{b_1^3+9b_3-4b_1b_2}{3b_2-b_1^2}, \quad -\lambda_{2,3}=\frac{b_1b_2-9b_3}{2\left(3b_2-b_1^2\right)}. \tag{S22}$$

(3) $D_2=0\wedge D_3=0$, there are three repeated real roots

$$-\lambda_{1,2,3}=-\sqrt[3]{b_3}. \tag{S23}$$

(4) $D_2=0\wedge D_3<0$, there are a real root and a pair of complex roots:

$$-\lambda_j=\frac{\sqrt[3]{9b_1b_2-27b_3-2b_1^3}}{3}\left[\cos\frac{2(j-1)\pi}{3}+i\sin\frac{2(j-1)\pi}{3}\right]-\frac{1}{3}b_1, \quad j=1,2,3 \tag{S24}$$

(5) $D_2\neq 0\wedge D_3<0$, there are a real root and a pair of complex roots:

$$
\begin{aligned}
-\lambda_1 &= -\frac{1}{3}\left(\frac{\sqrt[3]{2}D_2}{\sqrt[3]{E+3\sqrt{-3D_3}}}+\frac{\sqrt[3]{E+3\sqrt{-3D_3}}}{\sqrt[3]{2}}+b_1\right) \\
-\lambda_{2,3} &= \frac{1}{6}\left(\frac{\sqrt[3]{2}D_2}{\sqrt[3]{E+3\sqrt{-3D_3}}}+\frac{\sqrt[3]{E+3\sqrt{-3D_3}}}{\sqrt[3]{2}}-2b_1\right)\pm i\frac{\sqrt{3}}{6}\left(\frac{\sqrt[3]{E+3\sqrt{-3D_3}}}{\sqrt[3]{2}}-\frac{\sqrt[3]{2}D_2}{\sqrt[3]{E+3\sqrt{-3D_3}}}\right),
\end{aligned} \tag{S25}
$$

where $E=27b_3-9b_1b_2+2b_1^3$. In cases (1) to (5), the symbol '∧' denotes the 'and' operator in Boolean logic.

According to the above classification, we can show the explicit expression of initiation time distribution $f_{\text{ini}}(t)$ as follows

(1) For three district real roots $-\lambda_1,-\lambda_2,-\lambda_3$, the initiation time distribution is

$$
f_{\text{ini}}(t)=c_{11}e^{-\lambda_1 t}+c_{21}e^{-\lambda_2 t}+c_{31}e^{-\lambda_3 t}, \tag{S26}
$$

where $c_{11}=\dfrac{a_1\lambda_1^2-a_2\lambda_1+a_3}{(\lambda_2-\lambda_1)(\lambda_3-\lambda_1)},\quad c_{21}=\dfrac{a_1\lambda_2^2-a_2\lambda_2+a_3}{(\lambda_1-\lambda_2)(\lambda_3-\lambda_2)},\quad c_{31}=\dfrac{a_1\lambda_3^2-a_2\lambda_3+a_3}{(\lambda_1-\lambda_3)(\lambda_2-\lambda_3)}.$

(2) For three real roots with a simple root $-\lambda_1$ and two repeated roots $-\lambda_2=-\lambda_3$, the initiation time distribution is

$$
f_{\text{ini}}(t)=c_{11}e^{-\lambda_1 t}+c_{21}e^{-\lambda_2 t}+c_{22}te^{-\lambda_2 t}, \tag{S27}
$$

where $c_{11}=\dfrac{a_1\lambda_1^2-a_2\lambda_1+a_3}{(\lambda_2-\lambda_1)^2},\quad c_{21}=\dfrac{a_1\lambda_2^2-2a_1\lambda_1\lambda_2+a_2\lambda_1-a_3}{(\lambda_1-\lambda_2)^2},\quad c_{22}=\dfrac{a_1\lambda_2^2-a_2\lambda_2+a_3}{(\lambda_1-\lambda_2)}.$

(3) For three repeated real roots $-\lambda_1=-\lambda_2=-\lambda_3$, the initiation time distribution is

$$
f_{\text{ini}}(t)=c_{11}e^{-\lambda_1 t}+c_{12}te^{-\lambda_1 t}+c_{13}\frac{t^2}{2}e^{-\lambda_1 t}, \tag{S28}
$$

where $c_{11}=a_1,\quad c_{12}=a_2-2a_1\lambda_1,\quad c_{13}=a_1\lambda_1^2-a_2\lambda_1+a_3.$

(4) For a real root $-\lambda_1$ and a pair of complex roots $\lambda_{2,3}=-(\eta\pm \mathrm{i}\sigma)$, the initiation time distribution is

$$
f_{\text{ini}}(t)=c_{11}e^{-\lambda_1 t}+d_{11}e^{-\eta t}\cos(\sigma t)+e_{11}e^{-\eta t}\sin(\sigma t), \tag{S29}
$$

where

$$
c_{11}=\frac{a_1\lambda_1^2-a_2\lambda_1+a_3}{(\eta-\lambda_1)^2+\sigma^2},\quad d_{11}=\frac{a_1(\eta^2+\sigma^2)+(a_2-2a_1\eta)\lambda_1-a_3}{(\eta-\lambda_1)^2+\sigma^2},\quad e_{11}=\frac{(a_1\eta^2-a_2\eta+a_3)(\lambda_1-\eta)+(a_2-a_1\lambda_1-a_1\eta)\sigma^2}{\left((\eta-\lambda_1)^2+\sigma^2\right)\sigma}.
$$

C. The distribution of initiation times for arbitrary four-state model

For a four-state model with arbitrary promoter switching, the Laplace transform of initiation time distribution takes the following form

$$
\widetilde{f}_{\text{ini}}(s)=\frac{a_1s^3+a_2s^2+a_3s+a_4}{s^4+b_1s^3+b_2s^2+b_3s+b_4}. \tag{S30}
$$

To obtain the explicit expression of $f_{\text{ini}}(t)$, we first need to find the roots of the characteristic equation

$$s^4+b_1s^3+b_2s^2+b_3s+b_4=0. \tag{S31}$$

Set $s=\zeta-1/4b_1$, Eq. (S31) becomes

$$\zeta^4+f_1\zeta^2++f_2\zeta+f_3=0, \tag{S32}$$

where $f_1=b_2-3/8b_1^2$, $f_2=b_3-b_1b_2/2+b_1^3/8$, $f_3=b_4-b_1b_3/4+b_1^2b_2/16-3b_1^4/256$.

Furthermore, Eq. (S32) can be decomposed into the following form

$$\left(\zeta^2-g\zeta+h\right)\left(\zeta^2+g\zeta+l\right)=0, \tag{S33}$$

where $h=f_1/2+g^2/2+f_2/(2g)$, $l=f_1/2+g^2/2-f_2/(2g)$, $hl=f_3$. Eliminating h and l of Eq. (S33), we get

$$g^6+2f_1g^4+\left(f_1^2-4f_3\right)g^2-f_2^2=0, \tag{S34}$$

which is a cubic equation of g^2, and the roots can be found by using the method proposed in the above three-state model. Assume that g_1^2, g_2^2, and g_3^2 are the roots of Eq. (S34), then we have $g_1^2g_2^2g_3^2=f_2^2$, $g_1^2+g_2^2+g_3^2=-2f_1$. Choosing the negative signs of g_1, g_2, and g_3, and using the factor $\left(\zeta^2-g\zeta+h\right)=0$ or $\left(\zeta^2+g\zeta+l\right)=0$ of Eq. (S33) plus the expression $s=\zeta-1/4b_1$, we can obtain the roots of the characteristic equation (S31) as follows

$$\begin{aligned} -\lambda_1&=\frac{1}{2}(g_1+g_2+g_3)-\frac{b_1}{4}, & -\lambda_2&=\frac{1}{2}(g_1-g_2-g_3)-\frac{b_1}{4},\\ -\lambda_3&=\frac{1}{2}(-g_1+g_2-g_3)-\frac{b_1}{4}, & -\lambda_4&=\frac{1}{2}(-g_1-g_2+g_3)-\frac{b_1}{4}. \end{aligned} \tag{S35}$$

In addition, we can further give the initiation-time distribution according to the classification of characteristic roots [2].

Set

$$\begin{aligned} D_2&=3b_1^2-8b_2,\\ D_3&=16b_2b_4+14b_1b_2b_3+b_1^2b_2^2-3b_1^3b_3-6b_1^2b_4-4b_2^3-18b_3^2,\\ D_4&=256b_4^3-27b_3^4-192b_1b_3b_4^2-27b_1^4b_4^2-6b_1^2b_3^2b_4\\ &\quad+b_1^2b_2^2b_3^2-4b_2^3b_3^2+18b_1^3b_2b_3b_4+144b_1^2b_2b_4^2\\ &\quad-80b_1b_2^2b_3b_4+18b_1b_2b_3^3-4b_1^2b_2^3b_4-4b_1^3b_3^3\\ &\quad+16b_2^4b_4-128b_2^2b_4^2+144b_2b_3^2b_4.\\ E&=8b_3+b_1^3-4b_1b_2. \end{aligned} \tag{S36}$$

Then we have

(1) $D_4>0\wedge D_3>0\wedge D_2>0$, there are four district real roots $-\lambda_j\ (j=1,2,3,4)$, and the initiation time distribution

$$f_{\text{ini}}(t)=c_{11}e^{-\lambda_1t}+c_{21}e^{-\lambda_2t}+c_{31}e^{-\lambda_3t}+c_{41}e^{-\lambda_4t}. \tag{S37}$$

(2) $D_4 < 0$, there are two district real roots $-\lambda_j\,(j=1,2)$, and a pair of complex roots $-(\eta_1 \pm \mathrm{i}\sigma_1)$, and the initiation time distribution

$$f_{\text{ini}}(t) = \sum_{j=1}^{2} c_{j1} e^{-\lambda_j t} + d_{11} e^{-\eta_1 t} \cos(\sigma_1 t) + e_{11} e^{-\eta_1 t} \sin(\sigma_1 t). \tag{S38}$$

(3) $D_4 = 0 \wedge D_3 > 0$, there are four real roots with two simple roots $-\lambda_j\,(j=1,2)$, and two repeated roots. $-\lambda_3 = -\lambda_4$, and the initiation time distribution

$$f_{\text{ini}}(t) = \sum_{j=1}^{2} c_{j1} e^{-\lambda_j t} + c_{31} e^{-\lambda_3 t} + c_{32} t e^{-\lambda_3 t}. \tag{S39}$$

(4) $D_4 = 0 \wedge D_3 < 0$, there are two repeated real roots $-\lambda_1 = -\lambda_2$ and a pair of complex roots $-(\eta_1 \pm \mathrm{i}\sigma_1)$, and the initiation time distribution

$$f_{\text{ini}}(t) = c_{11} e^{-\lambda_1 t} + c_{12} t e^{-\lambda_1 t} + d_{11} e^{-\eta_1 t} \cos(\sigma_1 t) + e_{11} e^{-\eta_1 t} \sin(\sigma_1 t). \tag{S40}$$

(5) $D_4 = 0 \wedge D_3 = 0 \wedge D_2 > 0 \wedge E = 0$, there are two pairs of real repeated roots $-\lambda_1 = -\lambda_3$ and $-\lambda_2 = -\lambda_4$, and the initiation time distribution

$$f_{\text{ini}}(t) = c_{11} e^{-\lambda_1 t} + c_{12} t e^{-\lambda_1 t} + c_{21} e^{-\lambda_2 t} + c_{22} t e^{-\lambda_2 t}. \tag{S41}$$

(6) $D_4 = 0 \wedge D_3 = 0 \wedge D_2 > 0 \wedge E \neq 0$, there are four real roots with a simple root $-\lambda_1$, and three repeated roots $-\lambda_2 = -\lambda_3 = -\lambda_4$, and the initiation time distribution

$$f_{\text{ini}}(t) = c_{11} e^{-\lambda_1 t} + c_{21} e^{-\lambda_2 t} + c_{22} t e^{-\lambda_2 t} + c_{23} \frac{t^2}{2} e^{-\lambda_2 t}. \tag{S42}$$

(7) $D_4 = 0 \wedge D_3 = 0 \wedge D_2 = 0$, there are four repeated real roots $-\lambda_1 = -\lambda_2 = -\lambda_3 = -\lambda_4$, and the initiation time distribution

$$f_{\text{ini}}(t) = c_{11} e^{-\lambda_1 t} + c_{12} t e^{-\lambda_1 t} + c_{13} \frac{t^2}{2} e^{-\lambda_1 t} + c_{14} \frac{t^3}{6} e^{-\lambda_1 t}. \tag{S43}$$

In Eqs. (S37)-(S43), the real constants c_{jk} , d_{jk} with e_{jk} are obtained by using the same method as in Text S1.1.

D. The distribution of initiation times for the multi-state loop model

Here, we consider a multi-state model with a loop promoter structure, and derive the analytical expression for the initiation time distribution. In this model, the promoter progresses sequentially through several irreversible states, including $N-1$ inactive (OFF) states and one active (ON) state, forming a loop. The initiation matrix $\mathbf{A}_{\text{ini}}$ for the loop model has following form

$$\mathbf{A}_{\text{ini}}=\begin{pmatrix} -\lambda_{12} & & & & & \lambda_{N1} \\ \lambda_{12} & -\lambda_{23} & & & & 0 \\ & \lambda_{23} & -\lambda_{34} & & & 0 \\ & & \ddots & \ddots & & \vdots \\ & & & \lambda_{(N-2)(N-1)} & -\lambda_{(N-1)N} & 0 \\ & & & & \lambda_{(N-1)N} & -(\lambda_{N1}+\mu) \end{pmatrix}. \quad \text{(S44)}$$

Set $\det(s\mathbf{I}-\mathbf{A}_{\text{ini}})$ and $(s\mathbf{I}-\mathbf{A}_{\text{ini}})^*$ as the determinant and adjoint matrix of matrix $(s\mathbf{I}-\mathbf{A}_{\text{ini}})$, respectively. Substituting the inverse $(s\mathbf{I}-\mathbf{A}_{\text{ini}})^{-1}=(s\mathbf{I}-\mathbf{A}_{\text{ini}})^*/\det(s\mathbf{I}-\mathbf{A}_{\text{ini}})$ into $\tilde{f}_{\text{ini}}(s)=\mathbf{u}_{\text{ini}}(\text{s}\mathbf{I}-\mathbf{A}_{\text{ini}})^{-1}\mathbf{P}(0)$, and we obtain

$$\tilde{f}_{\text{ini}}(s)=\frac{a(s)}{b(s)}=\frac{\mu\prod_{j=1}^{N-1}\left(s+\lambda_{j(j+1)}\right)}{\left(s+\mu+\lambda_{N1}\right)\prod_{j=1}^{N-1}\left(s+\lambda_{j(j+1)}\right)-\lambda_{N1}\prod_{l=1}^{N-1}\lambda_{l(l+1)}}. \quad \text{(S45)}$$

It is easy to prove that the poles of $b(s)$ in Eq. (S45) have no repeated roots because the derivative of the characteristic polynomial of the initiation matrix $\mathbf{A}_{\text{ini}}$ is non-zero at the eigenvalues. In addition, the poles of $b(s)$ in Eq. (S45) can be complex. For example, for the forward transition rates $\lambda_{l(l+1)}=\lambda,\ l=1,2,\cdots N-1,$ and the initiation rate $\mu=\lambda-\lambda_{N1},$ then the poles can be analytically given by $\lambda_k=-\lambda+\sqrt[N]{\lambda_{N1}/\lambda}\cos\left(2(k-1)\pi/N\right)\lambda+\text{i}\sqrt[N]{\lambda_{N1}/\lambda}\sin\left(2(k-1)\pi/N\right)\lambda,\ k=1,2,\cdots,N.$ Taking the inverse Laplace transform of Eq. (S45), we obtain the following distribution of initiation times for the multi-state loop model

$$f_{\text{ini}}(t)=\sum_{j=1}^{n_1}c_je^{-\lambda_jt}+\sum_{j=1}^{n_2}d_je^{-\eta_jt}\cos\left(\sigma_jt\right)++\sum_{j=1}^{n_2}e_je^{-\eta_jt}\sin\left(\sigma_jt\right), \quad \text{(S46)}$$

which implies that the PDF of the initiation times has the forms of trigonometric function for the loop promoter structure and has the potential to exhibit non-monotonic behavior.

Section S2: Deriving the steady-state distribution of nascent RNA

First, we give the derivation of Eq. (3) in the main Text. Since the elongation time is fixed, the nascent RNA number at time t is equal to the total number of production events that occur within the time interval $(t-T,t]$. Assuming that m production events occur within this interval, and the corresponding production times satisfy $t-T<t_1<t_2<\cdots<t_m\le t$. We note that this stochastic process constitutes a delayed renewal process. Let $\tau_1=t_1-(t-T)$ denote the first arrival time, with its probability density function given by $f_1(\tau)$. For each subsequent inter-event interval $\tau_i=t_i-t_{i-1}(i=2,\cdots,m)$, the probability density function is $f_{ini}(\tau)$. Thus, when $m=0,$ no transcription initiation events occur within the interval, and the probability is given by the survival function of $f_1(\tau)$.

When $m \geq 1$, the total number of production events involves three processes: (1) The first initiation event occurs at t_1 with the probability $f_1(\tau_1)d\tau_1$; (2) The subsequent $m-1$ initiation events occur at $t_2, t_3, \cdots, t_m$ with the probability $f_{\text{ini}}(\tau_2)d\tau_2 f_{\text{ini}}(\tau_3)d\tau_3 \cdots f_{\text{ini}}(\tau_m)d\tau_m$; (3) No initiation events occur in the remaining time $t-t_m$ with the probability $S_{\text{ini}}(t-t_m)$. Based on the analysis, we can give the expression for the steady-state distribution of nascent RNA

$$P(M=m)=\begin{cases} \int_T^\infty f_1(\tau)d\tau, & m=0 \\ \left(f_1 * f_{\text{ini}}^{m-1} * S_{\text{ini}}\right)(T), & m \geq 1 \end{cases} \tag{S47}$$

where $f_{\text{ini}}^{m-1}(t)$ is the $m-1$ convolution of $f_{\text{ini}}(t)$. Next, we derive the expression of $f_1(t)$. Denote $R(t)=\langle M(t)\rangle$ the mean number of initiations up to t, which satisfies the following equation [3]

$$R(t)=F_1(t)+\int_0^t R(t-\tau)dF_{\text{ini}}(\tau), \tag{S48}$$

where $F_1(t)=\int_0^t f_1(\tau)d\tau$ and $F_{\text{ini}}(t)=\int_0^t f_{\text{ini}}(\tau)d\tau$ are the cumulative distribution functions of $f_1(t)$ and $f_{\text{ini}}(t)$, respectively. $R(t)$ is known as the renewal function in renewal theory. Because nascent RNA production events are mutually independent, the process $M(t)$ is stationary. So we can set $R(t)=\rho^{-1}t$ and substitute the expression into Eq. (S48), then simple calculation gives

$$F_1(t)=\frac{1}{\rho}\int_0^t\left(1-F_{\text{ini}}(\tau)\right)d\tau. \tag{S49}$$

Note that $F_1(\infty)=1$, so we can get $\rho=\int_0^\infty\left(1-F_{\text{ini}}(\tau)\right)d\tau$, i.e., the mean waiting time between successive initiation events. Taking the derivative of Eq. (S49), we can derive

$$f_1(t)=\frac{1-F_{\text{ini}}(t)}{\rho}. \tag{S50}$$

Furthermore, the Laplace transform of $f_1(t)$ is given by

$$\tilde{f}_1(s)=\frac{1-\tilde{f}_{\text{ini}}(s)}{\rho s}. \tag{S51}$$

Based on the above analysis, we now derive the analytical expressions of the steady-state nascent RNA distribution. We first derive the probability $P(M=0)$, which can be calculated according to the first formula of Eq. (S47). Taking the Laplace transform of the formula with respect to T yields

$$\tilde{P}(0,s)=\int_0^\infty P(M=0)e^{-sT}dT=\frac{1-\tilde{f}_1(s)}{s}=\frac{\rho s-1+\tilde{f}_{\text{ini}}(s)}{\rho s^2}. \tag{S52}$$

The mean initiation time can be calculated according to $\rho=-\left[d\tilde{f}_{\text{ini}}(s)/ds\right]\big|_{s=0}=\left(b_{N-1}-a_{N-1}\right)/b_N$. Substituting it and

Eq. (S2) into Eq. (S52), we get

$$\tilde{P}(0,s)=\frac{s^{N-1}+\vartheta_1 s^{N-2}+\cdots\vartheta_{N-2}s+\vartheta_{N-1}}{s^N+b_1s^{N-1}+b_2s^{N-2}+\cdots b_{N-1}s+b_N},\tag{S53}$$

where $\vartheta_1=b_1-b_N/(b_{N-1}-a_{N-1})$, $\vartheta_2=b_2+b_N(a_1-b_1)/(b_{N-1}-a_{N-1})$, $\cdots$, $\vartheta_{N-1}=b_{N-1}+b_N(a_{N-2}-b_{N-2})/(b_{N-1}-a_{N-1})$.

Taking the inverse Laplace transform of $\tilde{P}(0,s)$ in Eq. (S53) yields

$$P(M=0)=\sum_{j=1}^{n_1}\sum_{k=1}^{p_j}C_{j,k,0}\frac{T^{k-1}}{(k-1)!}e^{-\lambda_jT}+\sum_{j=1}^{n_2}\sum_{k=1}^{q_j}D_{j,k,0}\frac{T^{k-1}}{(k-1)!}e^{-\eta_jT}\cos(\sigma_jT)+\sum_{j=1}^{n_2}\sum_{k=1}^{q_j}E_{j,k,0}\frac{T^{k-1}}{(k-1)!}e^{-\eta_jT}\sin(\sigma_jT),\tag{S54}$$

where $C_{j,k,0}$, $D_{j,k,0}$ with $E_{j,k,0}$ are real constants and can be obtained using the same method in Text S1.1.

Next, we compute $P(M=m)(m\geq 1)$, which can be calculated according to the second formula of Eq. (S47). Taking the Laplace transform of the formula with respect to T yields

$$\tilde{P}(m,s)=\int_0^\infty P(M=m)e^{-sT}dT=\frac{\tilde{f}_1(s)\left(1-\tilde{f}_{\text{ini}}(s)\right)\left(\tilde{f}_{\text{ini}}(s)\right)^{m-1}}{s}.\tag{S55}$$

By simple calculation, and set $\Delta_j=b_j-a_j\,(j=1,2,\cdots,N-1)$ and $\bar{a}_j=a_j/a_1\,(j=2,3,\cdots,N)$, the Laplace transform $\tilde{P}(m,s)$ becomes

$$\tilde{P}(m,s)=\chi\frac{\left(s^{N-1}+\Delta_1s^{N-2}+\cdots+\Delta_{N-2}s+\Delta_{N-1}\right)^2\left(s^{N-1}+\bar{a}_2s^{N-2}+\cdots\bar{a}_{N-1}s+\bar{a}_N\right)^{m-1}}{\left(s^N+b_1s^{N-1}+b_2s^{N-2}+\cdots b_{N-1}s+b_N\right)^{m+1}},\tag{S56}$$

where $\chi=b_Na_1^{m-1}/(b_{N-1}-a_{N-1})$. For convenience, we rewrite $\tilde{P}(m,s)=\chi\bar{P}(m,s)$ with

$$\bar{P}(m,s)=\frac{(\Delta(s))^2\cdot(\bar{a}(s))^{m-1}}{(b(s))^{m+1}}=\frac{\left(s^{N-1}+\Delta_1s^{N-2}+\cdots+\Delta_{N-2}s+\Delta_{N-1}\right)^2\cdot\left(s^{N-1}+\bar{a}_2s^{N-2}+\cdots\bar{a}_{N-1}s+\bar{a}_N\right)^{m-1}}{\left(s^N+b_1s^{N-1}+b_2s^{N-2}+\cdots b_{N-1}s+b_N\right)^{m+1}}.\tag{S57}$$

The partial fraction decomposition of $\bar{P}(m,s)$ is given by

$$\bar{P}(m,s)=\sum_{j=1}^{n_1}\sum_{k=0}^{p_j(m+1)-1}\frac{\bar{C}_{j,k,m}}{(s+\lambda_j)^{p_j(m+1)-k}}+\sum_{j=1}^{n_2}\sum_{k=0}^{q_j(m+1)-1}\frac{\bar{D}_{j,k,m}+\mathrm{i}\bar{E}_{j,k,m}}{2(s+\eta_j+\mathrm{i}\sigma_j)^{q_j(m+1)-k}}+\sum_{j=1}^{n_2}\sum_{k=0}^{q_j(m+1)-1}\frac{\bar{D}_{j,k,m}-\mathrm{i}\bar{E}_{j,k,m}}{2(s+\eta_j-\mathrm{i}\sigma_j)^{q_j(m+1)-k}},\tag{S58}$$

where

$$\begin{aligned}
\bar{C}_{j,k,m}&=\frac{1}{k!}\frac{d^k}{ds^k}\left[\bar{P}(m,s)(s+\lambda_j)^{p_j(m+1)}\right]\Bigg|_{s=-\lambda_j},\\
\bar{D}_{j,k,m}&=\frac{1}{k!}\left\{\frac{d^k}{ds^k}\left[\bar{P}(m,s)(s+\eta_j+\mathrm{i}\sigma_j)^{q_j(m+1)}\right]\Bigg|_{s=-(\eta_j+\mathrm{i}\sigma_j)}+\frac{d^k}{ds^k}\left[\bar{P}(m,s)(s+\eta_j-\mathrm{i}\sigma_j)^{q_j(m+1)}\right]\Bigg|_{s=-(\eta_j-\mathrm{i}\sigma_j)}\right\},\\
\bar{E}_{j,k,m}&=-\frac{1}{k!}\mathrm{i}\left\{\frac{d^k}{ds^k}\left[\bar{P}(m,s)(s+\eta_j+\mathrm{i}\sigma_j)^{q_j(m+1)}\right]\Bigg|_{s=-(\eta_j+\mathrm{i}\sigma_j)}-\frac{d^k}{ds^k}\left[\bar{P}(m,s)(s+\eta_j-\mathrm{i}\sigma_j)^{q_j(m+1)}\right]\Bigg|_{s=-(\eta_j-\mathrm{i}\sigma_j)}\right\}.
\end{aligned}\tag{S59}$$

These coefficients can be found by using the same method as in Text S1.1. That is, we denote the possible real or

complex poles of $b(s)$ as $-x$ with multiplicity θ, more precisely , $x=\lambda_j,\theta=p_j$ for the real poles and $x=\eta_j\pm \mathrm{i}\sigma_j$, $\theta=q_j$ for the complex poles. In addition, the remain possible poles without considering multiplicities are denoted as $-x_1,-x_2,\cdots,-x_{N-\theta}$. Similarly, the possible poles of $\Delta(s)$ and $\bar{a}(s)$ in Eq. (S57) without considering multiplicities are denoted as $-z_1,-z_2,\cdots,-z_{N-1}$ and $-y_1,-y_2,\cdots,-y_{N-1}$, respectively. Set

$$w_l(s)=(s+z_l)^2,\quad l=1,2,\cdots,N-1 \tag{S60}$$

$$v_l(s)=\begin{cases}(s+y_l)^{m-1}/(s+x_l)^{m+1}, & l=1,\cdots,N-\theta\\ (s+y_l)^{m-1}. & l=N-\theta+1,\cdots,N-1\end{cases} \tag{S61}$$

Define two auxiliary functions $w(s)$ and $v(s)$

$$w(s)=\prod_{l=1}^{N-1}w_l(s),\quad v(s)=\prod_{l=1}^{N-1}v_l(s). \tag{S62}$$

Accordingly, the $n-$order derivative of $w(s)$ is

$$(w(s))^{(n)}=n!\sum_{r_1+\cdots r_{N-1}=n}\frac{(w_1(s))^{(r_1)}}{r_1!}\frac{(w_2(s))^{(r_2)}}{r_2!}\cdots\frac{(w_{N-1}(s))^{(r_{N-1})}}{r_{N-1}!}, \tag{S63}$$

with

$$(w_l(s))^{(r)}=\begin{cases}2(s+z_l),r=1\\ 2,\qquad r=2\\ 0,\ r=3,4,\cdots,n\end{cases}. \tag{S64}$$

The $n-$order derivative of $v(s)$ is

$$(v(s))^{(n)}=n!\sum_{r_1+\cdots r_{N-1}=n}\frac{(v_1(s))^{(r_1)}}{r_1!}\frac{(v_2(s))^{(r_2)}}{r_2!}\cdots\frac{(v_{N-1}(s))^{(r_{N-1})}}{r_{N-1}!}, \tag{S65}$$

with

$$(v_l(s))^{(r)}=\begin{cases}(-m-1)_r\dfrac{(s+y_l)^{m-1}}{(s+x_l)^{m+1+r}}\,{}_2F_1\left(-r,1-m;-r-m;\dfrac{s+x_l}{s+y_l}\right), & l=1,2,\cdots,N-\theta\\ (-1)^r(y_l)^{m-1-r}(1-m)_r\,{}_2F_1\left(1-m+r,\kappa+r;\kappa+r;-\dfrac{s}{y_l}\right), & l=N-\theta+1,\cdots,N-1\end{cases} \tag{S66}$$

Substituting Eq. (S60)–(S66) and $s=-x$ into Eq. (S59), we can find the following coefficients

$$\bar{C}_{j,k,m}=\begin{cases}\left.\sum_{h=0}^{k}\dfrac{1}{h!(k-h)!}w^{(h)}(s)v^{k-h}(s)\right|_{s=-\lambda_j}, & k=0,1,\cdots,\min\{2(N-1)-1,p_j(m+1)-1\}\\ \left.\sum_{h=0}^{2(N-1)}\dfrac{1}{h!(k-h)!}w^{(h)}(s)v^{k-h}(s)\right|_{s=-\lambda_j}, & k=2(N-1),2(N-1)+1,\cdots,p_j(m+1)-1\end{cases} \tag{S67}$$

$$\bar{D}_{j,k,m}=\begin{cases}\sum_{h=0}^{k}\frac{1}{h!(k-h)!}\left(w^{(h)}(s)v^{k-h}(s)\Big|_{s=-(\eta_j+\mathrm{i}\sigma_j)}+w^{(h)}(s)v^{k-h}(s)\Big|_{s=-(\eta_j-\mathrm{i}\sigma_j)}\right), k=0,1,\cdots,\min\{2(N-1)-1,q_j(m+1)-1\}\\ \sum_{h=0}^{2(N-1)}\frac{1}{h!(k-h)!}\left(w^{(h)}(s)v^{k-h}(s)\Big|_{s=-(\eta_j+\mathrm{i}\sigma_j)}+w^{(h)}(s)v^{k-h}(s)\Big|_{s=-(\eta_j-\mathrm{i}\sigma_j)}\right), k=2(N-1),2(N-1)+1,\cdots,q_j(m+1)-1\end{cases}\tag{S68}$$

$$\bar{E}_{j,k,m}=\begin{cases}\sum_{h=0}^{k}-\mathrm{i}\frac{1}{h!(k-h)!}\left(w^{(h)}(s)v^{k-h}(s)\Big|_{s=-(\eta_j+\mathrm{i}\sigma_j)}-w^{(h)}(s)v^{k-h}(s)\Big|_{s=-(\eta_j-\mathrm{i}\sigma_j)}\right), k=0,1,\cdots,\min\{2(N-1)-1,q_j(m+1)-1\}\\ \sum_{h=0}^{2(N-1)}-\mathrm{i}\frac{1}{h!(k-h)!}\left(w^{(h)}(s)v^{k-h}(s)\Big|_{s=-(\eta_j+\mathrm{i}\sigma_j)}-w^{(h)}(s)v^{k-h}(s)\Big|_{s=-(\eta_j-\mathrm{i}\sigma_j)}\right), k=2(N-1),2(N-1)+1,\cdots,q_j(m+1)-1\end{cases}\tag{S69}$$

To obtain the inverse Laplace transform of $\bar{P}(n,s)$, we rewrite Eq. (S58) as follows

$$\begin{aligned}\bar{P}(m,s)=&\sum_{j=1}^{n_1}\sum_{k=0}^{p_j(m+1)-1}\frac{\bar{C}_{j,k,m}}{(s+\lambda_j)^{p_j(m+1)-k}}+\sum_{j=1}^{n_2}\sum_{k=0}^{q_j(m+1)-1}\bar{D}_{j,k,m}\left(\frac{1}{2(s+\eta_j+\sigma_j)^{q_j(m+1)-k}}+\frac{1}{2(s+\eta_j-\sigma_j)^{q_j(m+1)-k}}\right)\\&+\sum_{j=1}^{n_2}\sum_{k=0}^{q_j(m+1)-1}\bar{E}_{j,k,m}\mathrm{i}\left(\frac{1}{2(s+\eta_j+\sigma_j)^{q_j(m+1)-k}}-\frac{1}{2(s+\eta_j-\sigma_j)^{q_j(m+1)-k}}\right).\end{aligned}\tag{S70}$$

Taking the inverse Laplace transform of Eq. (S70) with the identities in Eq. (S11), and denote $C_{j,k,m}=\chi\bar{C}_{j,k,m}$, $D_{j,k,m}=\chi\bar{D}_{j,k,m}$, $E_{j,k,m}=\chi\bar{E}_{j,k,m}$, we get

$$P(M=m)=\sum_{j=1}^{n_1}\sum_{k=0}^{p_j(m+1)-1}\frac{C_{j,k,m}T^{p_j(m+1)-1-k}e^{-\lambda_jT}}{(p_j(m+1)-1-k)!}+\sum_{j=1}^{n_2}\sum_{k=0}^{q_j(m+1)-1}\frac{T^{q_j(m+1)-1-k}e^{-\eta_jT}}{(q_j(m+1)-1-k)!}\left(D_{j,k,m}\cos(\sigma_jT)+E_{j,k,m}\sin(\sigma_jT)\right).\tag{S71}$$

In particular, we give the analytical steady-state distributions of nascent RNA for three-state models with arbitrary promoter switching, which have the following four cases according to the classification of the roots of the equation $s^3+b_1s^2+b_2s+b_3=0$ in Text S1.2B.

(1) For three district real roots $-\lambda_1,-\lambda_2,-\lambda_3$, we get

$$P(M=m)=\begin{cases}C_{1,1,m}e^{-\lambda_1T}+C_{2,1,m}e^{-\lambda_2T}+C_{3,1,m}e^{-\lambda_3T}, & m=0\\ \sum_{k=0}^{m}\frac{T^{m-k}\left(C_{1,k,m}e^{-\lambda_1T}+C_{2,k,m}e^{-\lambda_2T}+C_{3,k,m}e^{-\lambda_3T}\right)}{(m-k)!}, & m\ge1\end{cases}\tag{S72}$$

(2) For three real roots with a simple root $-\lambda_1$ and two repeated roots $-\lambda_2=-\lambda_3$, we get

$$P(M=m)=\begin{cases}C_{1,1,m}e^{-\lambda_1T}+C_{2,1,m}e^{-\lambda_2T}+C_{2,2,m}te^{-\lambda_2T}, & m=0\\ \sum_{k=0}^{m}\frac{C_{1,k,m}T^{m-k}e^{-\lambda_1T}}{(m-k)!}+\sum_{k=0}^{2m+1}\frac{C_{2,k,m}T^{2m+1-k}e^{-\lambda_2T}}{(2m+1-k)!}, & m\ge1\end{cases}\tag{S73}$$

(3) For three repeated real roots $-\lambda_1=-\lambda_2=-\lambda_3$, we get

$$P(M=m)=\begin{cases} C_{1,1,m}e^{-\lambda_1 T}+C_{1,2,m}Te^{-\lambda_1 T}+C_{1,3,m}\dfrac{T^2}{2}e^{-\lambda_1 T}, & m=0 \\ \displaystyle\sum_{k=0}^{3m+2}\frac{C_{1,k,m}T^{3m+2-k}e^{-\lambda_1 T}}{(3m+2-k)!}, & m\geq 1 \end{cases} \tag{S74}$$

(4) For a real root $-\lambda_1$ and a pair of conjugate complex roots $-\lambda_{2,3}=-(\eta\pm i\sigma)$, we get

$$P(M=m)=\begin{cases} C_{1,1,m}e^{-\lambda_1 T}+D_{1,1,m}e^{-\eta T}\cos(\sigma T)+E_{1,1,m}e^{-\eta T}\sin(\sigma T), & m=0 \\ \displaystyle\sum_{k=0}^{m}\frac{T^{m-k}\left(C_{1,k,m}e^{-\lambda_1 T}+D_{1,k,m}e^{-\eta T}\cos(\sigma T)+E_{1,k,m}e^{-\eta T}\sin(\sigma T)\right)}{(m-k)!}, & m\geq 1 \end{cases} \tag{S75}$$

Section S3: Deriving the binomial moments of steady-state nascent RNA distribution

In this section, we derive the steady-state binomial moments B_k of nascent RNA distribution. We denote $G(z)=\sum_{m=0}^{\infty}z^m P(M=m)$ as the probability generating function of $P(M=m)$, whose Laplace transform $\tilde{G}(z,s)$ is given by $\tilde{G}(z,s)=1/s+\tilde{f}_1(s)(z-1)\big/\left(s-zs\tilde{f}_{\text{ini}}(s)\right)$. The Laplace transform of binomial moments B_k can be obtained according to $\tilde{B}_k(s)=1/k!d^k\tilde{G}(z,s)/dz^k\big|_{z=1}$, which has the following form of rational function

$$\tilde{B}_k(s)=b_N\big/(b_{N-1}-a_{N-1})s^{k-3}\left(\tilde{R}(s)\right)^{k-1}, \tag{76}$$

where $\tilde{R}(s)=\tilde{f}_{\text{ini}}(s)\big/\left(s-s\tilde{f}_{\text{ini}}(s)\right)$.

Set $\Upsilon=b_N\big/(b_{N-1}-a_{N-1})$, substitute Eq. (S2) into $\tilde{R}(s)$, and then we rewrite $\tilde{B}_k(s)=\Upsilon\bar{B}_k(s)$ with

$$\bar{B}_k(s)=\frac{(a(s))^{k-1}}{s^{k+1}(\Delta(s))^{k-1}}=\frac{\left(a_1s^{N-1}+a_2s^{N-2}+\cdots+a_{N-1}s+a_N\right)^{k-1}}{s^{k+1}\left(s^{N-1}+\Delta_1s^{N-2}+\cdots+\Delta_{N-2}s+\Delta_{N-1}\right)^{k-1}}. \tag{S77}$$

Using the same method in Text S1.1, we can give the partial fraction decomposition of $\bar{B}_k(s)$ as follows

$$\begin{aligned}\bar{B}_k(s)=&\sum_{j=1}^{k+1}\frac{\bar{F}_{j,k}}{s^j}+\sum_{j=1}^{n_1^\Delta}\sum_{h=0}^{p_j^\Delta(k-1)-1}\frac{\bar{G}_{j,h,k}}{\left(s+\lambda_j^\Delta\right)^{p_j^\Delta(k-1)-h}}+\sum_{j=1}^{n_2^\Delta}\sum_{h=0}^{q_j^\Delta(k-1)-1}\frac{\bar{H}_{j,h,k}+\mathrm{i}\bar{K}_{j,h,k}}{2\left(s+\eta_j^\Delta+\mathrm{i}\sigma_j^\Delta\right)^{q_j^\Delta(k-1)-h}}\\&+\sum_{j=1}^{n_2^\Delta}\sum_{h=0}^{q_j^\Delta(k-1)-1}\frac{\bar{H}_{j,h,k}-\mathrm{i}\bar{K}_{j,h,k}}{2\left(s+\eta_j^\Delta-\mathrm{i}\sigma_j^\Delta\right)^{q_j^\Delta(k-1)-h}},\end{aligned} \tag{S78}$$

with

$$\begin{aligned}&\bar{F}_{j,k}=\frac{1}{(k+1-j)!}\frac{d^{(k+1-j)}}{ds^{(k+1-j)}}\left[\bar{B}_k(s)s^{k+1}\right]\Bigg|_{s=0},\quad \bar{G}_{j,h,k}=\frac{1}{h!}\frac{d^h}{ds^h}\left[\bar{B}_k(s)\left(s+\lambda_j^\Delta\right)^{p_j^\Delta(k-1)}\right]\Bigg|_{s=-\lambda_j^\Delta},\\&\bar{H}_{j,h,k}=\frac{1}{h!}\left\{\frac{d^h}{ds^h}\left[\bar{B}_k(s)\left(s+\eta_j^\Delta+\mathrm{i}\sigma_j^\Delta\right)^{q_j^\Delta(k-1)}\right]\Bigg|_{s=-\left(\eta_j^\Delta+\mathrm{i}\sigma_j^\Delta\right)}+\frac{d^h}{ds^h}\left[\bar{B}_k(s)\left(s+\eta_j^\Delta-\mathrm{i}\sigma_j^\Delta\right)^{q_j^\Delta(k-1)}\right]\Bigg|_{s=-\left(\eta_j^\Delta-\mathrm{i}\sigma_j^\Delta\right)}\right\},\\&\bar{K}_{j,h,k}=-\frac{1}{h!}\mathrm{i}\left\{\frac{d^h}{ds^h}\left[\bar{B}_k(s)\left(s+\eta_j^\Delta+\mathrm{i}\sigma_j^\Delta\right)^{q_j^\Delta(k-1)}\right]\Bigg|_{s=-\left(\eta_j^\Delta+\mathrm{i}\sigma_j^\Delta\right)}-\frac{d^h}{ds^h}\left[\bar{B}_k(s)\left(s+\eta_j^\Delta-\mathrm{i}\sigma_j^\Delta\right)^{q_j^\Delta(k-1)}\right]\Bigg|_{s=-\left(\eta_j^\Delta-\mathrm{i}\sigma_j^\Delta\right)}\right\}.\end{aligned} \tag{S79}$$

To find the coefficient $\bar{F}_{j,k}$, we denote the poles of $\Delta(s)$ and $a(s)$ in Eq. (S77) without considering multiplicities as $-z_1,-z_2,\cdots,-z_{N-1}$ and $-y_1,-y_2,\cdots,-y_{N-1}$, respectively. Set

$$u_l(s)=(s+y_l)^{k-1}/(s+z_l)^{k-1},\quad l=1,2,\cdots,N-1 \tag{S80}$$

Define auxiliary function $u(s)=\prod_{l=1}^{N-1}u_l(s)$. Accordingly, the $n-$order derivative of $u(s)$ is

$$(u(s))^{(n)}=n!\sum_{r_1+\cdots r_{N-1}=n}\frac{(u_1(s))^{(r_1)}}{r_1!}\frac{(u_2(s))^{(r_2)}}{r_2!}\cdots\frac{(u_{N-1}(s))^{(r_{N-1})}}{r_{N-1}!}, \tag{S81}$$

with

$$(u_l(s))^{(r)}=(1-k)_r\frac{(s+y_l)^{k-1}}{(s+z_l)^{k-1+r}}\,{}_2F_1\left(-r,1-k;2-k-r;\frac{s+z_l}{s+y_l}\right),\quad r=1,2,\cdots,N-1 \tag{S82}$$

Substituting Eqs. (S80)–(S82) and $s=0$ into the first row of Eq. (S79), we can find the coefficient

$$\bar{F}_{j,k}=\left.\frac{(u(s))^{(k+1-j)}}{(k+1-j)!}\right|_{s=0}. \tag{S83}$$

These remain coefficients can be found by using the method discussed in Text S1.1. That is, we denote the possible real or complex poles of $\Delta(s)$ as $-z$ with multiplicity θ, more precisely , $z=\lambda_j^{\Delta},\theta=p_j^{\Delta}$ for the real poles and $z=\eta_j^{\Delta}\pm\mathrm{i}\sigma_j^{\Delta},\ \theta=q_j^{\Delta}$ for the complex poles. In addition, the remain possible poles without considering multiplicities are denoted as $-z_1,-z_2,\cdots,-z_{N-1-\theta}$. Similarly, the possible poles of $a(s)$ in Eq. (S77) without considering multiplicities are denoted as $-y_1,-y_2,\cdots,-y_{N-1}$, respectively. Set

$$v_l(s)=\begin{cases}(s+y_l)^{k-1}/(s+z_l)^{k-1}, & l=1,\cdots,N-1-\theta\\ (s+y_l)^{k-1}. & l=N-\theta,\cdots,N-1\end{cases} \tag{S84}$$

Define two auxiliary functions $w(s)$ and $v(s)$

$$w(s)=\frac{1}{s^{k+1}},\quad v(s)=\prod_{l=1}^{N-1}v_l(s). \tag{S85}$$

Accordingly, the $n-$order derivative of $w(s)$ is

$$(w(s))^{(n)}=(-1)^n(k+1)_n\,s^{-(k+1+n)}. \tag{S86}$$

The $n-$order derivative of $v(s)$ is

$$(v(s))^{(n)}=n!\sum_{r_1+\cdots r_{N-1}=n}\frac{(v_1(s))^{(r_1)}}{r_1!}\frac{(v_2(s))^{(r_2)}}{r_2!}\cdots\frac{(v_{N-1}(s))^{(r_{N-1})}}{r_{N-1}!}, \tag{S87}$$

with

$$
\left(v_l(s)\right)^{(r)}=\begin{cases}(1-k)_r\dfrac{(s+y_l)^{k-1}}{(s+z_l)^{k-1+r}}\,{}_2F_1\left(-r,1-k;2-k-r;\dfrac{s+z_l}{s+y_l}\right), & r=1,2,\cdots,N-1-\theta\\ (-1)^r(y_l)^{k-1-r}(1-k)_r\,{}_2F_1\left(1-k+r,\kappa+r;\kappa+r;-\dfrac{s}{y_l}\right), & r=N-\theta,\cdots,N-1.\end{cases} \tag{S88}
$$

Substituting Eqs. (S84)–(S88) and $s=-x$ into Eq. (S79), we can find the following coefficients

$$
\begin{aligned}
\bar{G}_{j,h,k}&=\sum_{l=0}^{h}\frac{1}{l!(h-l)!}w^{(l)}(s)v^{h-l}(s)\Big|_{s=-\lambda_j}, \quad h=0,1,\cdots,p_j^{\Delta}(k-1)-1,\\
\bar{H}_{j,h,k}&=\sum_{l=0}^{h}\frac{1}{l!(h-l)!}\left(w^{(l)}(s)v^{h-l}(s)\Big|_{s=-(\eta_j+\mathrm{i}\sigma_j)}+w^{(l)}(s)v^{h-l}(s)\Big|_{s=-(\eta_j-\mathrm{i}\sigma_j)}\right), \quad h=0,1,\cdots,q_j^{\Delta}(k-1)-1,\\
\bar{K}_{j,h,k}&=\sum_{l=0}^{h}-\frac{1}{l!(h-l)!}\mathrm{i}\left(w^{(l)}(s)v^{h-l}(s)\Big|_{s=-(\eta_j+\mathrm{i}\sigma_j)}-w^{(l)}(s)v^{h-l}(s)\Big|_{s=-(\eta_j-\mathrm{i}\sigma_j)}\right), \quad k=0,1,\cdots,q_j^{\Delta}(k-1)-1.
\end{aligned} \tag{S89}
$$

To the obtain the inverse Laplace transform of $\bar{B}_k(s)$, we rewrite Eq. (S78) as follows

$$
\begin{aligned}
\bar{B}_k(s)=&\sum_{j=1}^{k+1}\frac{\bar{F}_{j,k}}{s^j}+\sum_{j=1}^{n_1^{\Delta}}\sum_{h=0}^{p_j^{\Delta}(k-1)-1}\frac{\bar{G}_{j,h,k}}{\left(s+\lambda_j^{\Delta}\right)^{p_j^{\Delta}(k-1)-h}}\\
&+\sum_{j=1}^{n_2^{\Delta}}\sum_{h=0}^{q_j^{\Delta}(k-1)-1}\bar{H}_{j,h,k}\left(\frac{1}{2\left(s+\eta_j^{\Delta}+\mathrm{i}\sigma_j^{\Delta}\right)^{q_j^{\Delta}(k-1)-h}}+\frac{1}{2\left(s+\eta_j^{\Delta}-\mathrm{i}\sigma_j^{\Delta}\right)^{q_j^{\Delta}(k-1)-h}}\right)\\
&+\sum_{j=1}^{n_2^{\Delta}}\sum_{h=0}^{q_j^{\Delta}(k-1)-1}\bar{K}_{j,h,k}\mathrm{i}\left(\frac{1}{2\left(s+\eta_j^{\Delta}-\mathrm{i}\sigma_j^{\Delta}\right)^{q_j^{\Delta}(k-1)-h}}-\frac{1}{2\left(s+\eta_j^{\Delta}-\mathrm{i}\sigma_j^{\Delta}\right)^{q_j^{\Delta}(k-1)-h}}\right).
\end{aligned} \tag{S90}
$$

Taking the inverse Laplace transform of Eq. (S90) with the identities (S11), and denoting $F_{j,k}=\Upsilon\bar{F}_{j,k}$, $G_{j,l,k}=\Upsilon\bar{G}_{j,l,k}$, $H_{j,l,k}=\Upsilon\bar{H}_{j,l,k}$, $K_{j,l,k}=\Upsilon\bar{K}_{j,l,k}$, we get the moments

$$
\begin{aligned}
B_k=&\sum_{j=1}^{k+1}\frac{F_{j,k}}{(j-1)!}T^{j-1}+\sum_{j=1}^{n_1^{\Delta}}\sum_{h=0}^{p_j^{\Delta}(k-1)-1}\frac{G_{j,h,k}T^{p_j^{\Delta}(k-1)-1-h}e^{-\lambda_j^{\Delta}T}}{\left(p_j^{\Delta}(k-1)-1-h\right)!}\\
&+\sum_{j=1}^{n_2^{\Delta}}\sum_{h=0}^{q_j^{\Delta}(k-1)-1}\frac{T^{q_j^{\Delta}(k-1)-1-h}e^{-\eta_j^{\Delta}T}}{\left(q_j^{\Delta}(k-1)-1-h\right)!}\left(H_{j,h,k}\cos\left(\sigma_j^{\Delta}T\right)+K_{j,h,k}\sin\left(\sigma_j^{\Delta}T\right)\right).
\end{aligned} \tag{S91}
$$

In particular, we can calculate the central moments with the binomial moments

$$
B_k^c=(-B_1)^k+\sum_{i=0}^{k-1}\sum_{j=0}^{k-i}L(k,i,j)(j!)(B_1)^iB_j, \tag{92}
$$

where $L(k,i,j)=(-1)^i\binom{k}{i}S(k-i,j)$ with $S(n,k)=\sum_{i=0}^{k}(-1)^{k-i}\binom{k}{i}i^n$ being the Stirling number of the second kind. Therefore, the skewness $S=B_3^c/\left(B_2^c\right)^{3/2}$ and kurtosis $K=B_4^c/\left(B_2^c\right)^2$ can also be obtained. In addition, through simple calculation, the mean and noise of nascent RNA are given by

$$
\langle\mathrm{RNA}\rangle=b_NT/(b_{N-1}-a_{N-1}), \quad \eta_{\mathrm{RNA}}^2=\frac{(b_{N-1}-a_{N-1})\left(2\int_0^TR(t)dt+T\right)-b_NT^2}{b_NT^2}, \tag{93}
$$

where $R(t)$ is the inverse Laplace transform of $\tilde{R}(s)$.

Section S4: Identifying the kinetic parameters for several representative models

The kinetic parameters of models can be calculated according to the coefficients of Laplace transform of initiation-time distribution in Eq. (S2) and the steady-state binomial moment of nascent RNA distribution in Eq. (S91). For convenience, we denote X as nascent RNA and Y as mature RNA in the following initiation mechanisms.

S4.1: Identifying the kinetic parameters for the ON-OFF model

The reaction scheme for the ON-OFF model is given by

$$\mathrm{OFF}\xrightarrow{k_{\mathrm{on}}}\mathrm{ON}\xrightarrow{k_{\mathrm{off}}}\mathrm{OFF},\quad \mathrm{ON}\xrightarrow{\mu}\mathrm{ON}+\mathrm{X},\quad \mathrm{X}\overset{T}{\Rightarrow}Y. \tag{S94}$$

The Laplace transform of the initiation-time distribution for the ON-OFF model is

$$\tilde{f}_{\mathrm{ini}}(s)=\frac{a_1 s+a_2}{s^2+b_1 s+b_2}, \tag{S95}$$

where the coefficients $a_j, b_j\ (j=1,2)$ satisfy the following expressions

$$\mu=a_1,\quad k_{\mathrm{on}}\mu=a_2,\quad k_{\mathrm{on}}+k_{\mathrm{off}}=b_1-a_1. \tag{S96}$$

It is easy to obtain the kinetic parameters

$$\mu=a_1,\quad k_{\mathrm{on}}=a_2/a_1,\quad k_{\mathrm{off}}=b_1-a_1-a_2/a_1. \tag{S97}$$

In addition, the elongation time is given by

$$T=B_1(b_1-a_1)/b_2, \tag{S98}$$

where B_1 is the first-order binomial moment of the steady-state nascent RNA distribution.

S4.2: Identifying the kinetic parameters for the multi-state loop models

A. Three-state loop model

The reaction scheme for the three-state loop model is given by

$$\mathrm{OFF}_1\xrightarrow{\lambda_{12}}\mathrm{OFF}_2\xrightarrow{\lambda_{23}}\mathrm{ON}\xrightarrow{\lambda_{31}}\mathrm{OFF}_1,\quad \mathrm{ON}\xrightarrow{\mu}\mathrm{ON}+\mathrm{X},\quad \mathrm{X}\overset{T}{\Rightarrow}Y. \tag{S99}$$

The Laplace transform of the initiation-time distribution for the three-state loop model is

$$\tilde{f}_{\mathrm{ini}}(s)=\frac{a_1 s^2+a_2 s+a_3}{s^3+b_1 s^2+b_2 s+b_3}, \tag{S100}$$

where the coefficients $a_j, b_j\ (j=1,2,3)$ satisfy the following expressions

$$\begin{aligned}&\mu=a_1,\quad (\lambda_{12}+\lambda_{23})\mu=a_2,\quad \lambda_{12}\lambda_{23}\mu=a_3=b_3,\\ &\lambda_{12}+\lambda_{23}+\lambda_{31}=b_1-a_1,\quad (\lambda_{23}+\lambda_{31})\lambda_{12}+\lambda_{23}\lambda_{31}=b_2-a_2.\end{aligned} \tag{S101}$$

It is easy to find that λ_{12} and λ_{23} are the roots of equation

$$a_1x^2 - a_2x + a_3 = 0, \tag{S102}$$

which yields

$$\lambda_{12/23} = \frac{a_2 \pm \sqrt{a_2^2 - 4a_1a_3}}{2a_1}. \tag{S103}$$

The remaining parameters are given by

$$\mu = a_1, \quad \lambda_{31} = b_1 - a_1 - a_2/a_1. \tag{S104}$$

In addition, the elongation time is given by

$$T = B_1(b_2 - a_2)/b_3. \tag{S105}$$

B. Four-state loop model

The reaction scheme for the four-state loop model is given by

$$\mathrm{OFF}_1 \xrightarrow{\lambda_{12}} \mathrm{OFF}_2 \xrightarrow{\lambda_{23}} \mathrm{OFF}_3 \xrightarrow{\lambda_{34}} \mathrm{ON}, \quad \mathrm{ON} \xrightarrow{\lambda_{41}} \mathrm{OFF}_1, \quad \mathrm{ON} \xrightarrow{\mu} \mathrm{ON} + \mathrm{X}, \quad \mathrm{X} \overset{T}{\Rightarrow} Y. \tag{S106}$$

The Laplace transform of the initiation-time distribution for the four-state loop model is

$$\tilde{f}_{\text{ini}}(s) = \frac{a_1s^3 + a_2s^2 + a_3s + a_4}{s^4 + b_1s^3 + b_2s^2 + b_3s + b_4}, \tag{S107}$$

where the coefficients a_j, b_j $(j = 1,2,3,4)$ satisfy the following expressions

$$\begin{aligned} &\mu = a_1, \qquad (\lambda_{12} + \lambda_{23} + \lambda_{34})\mu = a_2, \qquad [(\lambda_{12} + \lambda_{23})\lambda_{34} + \lambda_{12}\lambda_{23}]\mu = a_3, \\ &\lambda_{12}\lambda_{23}\lambda_{34}\mu = a_4 = b_4, \qquad \lambda_{12} + \lambda_{23} + \lambda_{34} + \lambda_{41} = b_1 - a_1, \\ &(\lambda_{12} + \lambda_{23})\lambda_{34} + (\lambda_{12} + \lambda_{23} + \lambda_{34})\lambda_{41} + \lambda_{12}\lambda_{23} = b_2 - a_2, \\ &(\lambda_{34} + \lambda_{41})\lambda_{12}\lambda_{23} + (\lambda_{12} + \lambda_{23})\lambda_{34}\lambda_{41} = b_3 - a_3. \end{aligned} \tag{S108}$$

It is easy to find that λ_{12}, λ_{23} and λ_{34} are the real roots of cubic equation

$$a_1y^3 - a_2y^2 + a_3y - a_4 = 0, \tag{S109}$$

which can be obtained according to formula shown in Text S1.2. The remain parameters are given by

$$\mu = a_1, \qquad \lambda_{41} = b_1 - a_1 - a_2/a_1. \tag{S110}$$

In addition, the elongation time is given by

$$T = B_1(b_3 - a_3)/b_4. \tag{S111}$$

C. Five-state loop model

The reaction scheme for the five-state loop model is given by

$$\begin{aligned} &\mathrm{OFF}_1 \xrightarrow{\lambda_{12}} \mathrm{OFF}_2 \xrightarrow{\lambda_{23}} \mathrm{OFF}_3 \xrightarrow{\lambda_{34}} \mathrm{OFF}_4 \xrightarrow{\lambda_{45}} \mathrm{ON}, \\ &\mathrm{ON} \xrightarrow{\lambda_{51}} \mathrm{OFF}_1, \quad \mathrm{ON} \xrightarrow{\mu} \mathrm{ON} + \mathrm{X}, \quad \mathrm{X} \overset{T}{\Rightarrow} Y. \end{aligned} \tag{S112}$$

The Laplace transform of the initiation-time distribution for the five-state loop model is

$$\tilde{f}_{\text{ini}}(s)=\frac{a_1 s^4+a_2 s^3+a_3 s^2+a_4 s+a_5}{s^5+b_1 s^4+b_2 s^3+b_3 s^2+b_4 s+b_5}. \tag{S113}$$

Based on the above analysis, it is easy to find that λ_{12}, λ_{23}, λ_{34} and λ_{45} are the real roots of quartic equation

$$a_1 y^4-a_2 y^3+a_3 y^2-a_4 y+a_5=0, \tag{S114}$$

which can be obtained according to formula shown in Text S1.2. The remain parameters are given by

$$\mu=a_1,\qquad \lambda_{51}=b_1-a_1-a_2/a_1. \tag{S115}$$

In addition, the elongation time is given by

$$T=B_1(b_4-a_4)/b_5. \tag{S116}$$

Previous studies have shown that the theoretical limitations of inference from static gene expression data, e.g., the transition rates from one inactive state to another or to the active state are interchangeable [4]. Here our results obtained in Eqs. (S102), (S109), and (S114) provide an explanation for the interchangeability of these rate constants.

Section S5: Description of deconvolution algorithm

The deconvolution process aims to extract the time point of a single transcription initiation event from signals transcribed simultaneously by multiple RNA polymerases [5]. The transcription initiation events or RNAP II positions are found by using a genetic algorithm followed by a local optimization procedure.

First, the MS2 signal from one transcription site is modeled as

$$s(t)=\sum_{i=1}^{N_{\text{pol}}} s_{\text{pol}}(t-t_i), \tag{S117}$$

where s_{pol} is the signal from one Pol II and t_i are the successive initiation times, N_{pol} is the number of Pol II contributing to the signal. N_{pol} is not known and will be determined by the optimization procedure. The polymerase dwell time on the DNA is calculated using the single polymerase pattern according to $(l_{\text{pre}}+l_{\text{seq}}+l_{\text{post}})/V_{\text{pol}}+t_{\text{pol}}$, where l_{pre}, l_{seq} and l_{post} represent the length in base pairs of the three sequences: before the MS2 sequence, the MS2 sequence and the sequence after MS2, respectively; V_{pol} and t_{pol} are the elongation speed of the polymerase and polyadenylation time, respectively. Note that transcription begins when RNA polymerase II leaves the promoter. However, no signal will be generated before the MS2 sequence. During the transcription process of MS2 sequence, the signal can be represented as a linear increase. After MS2, the signal will remain constant until the polymerase leaves the transcription site and the signal suddenly decreases.

Second, the initiation times are discretized $t_i=n_i\delta$, where $n_i\in\mathbb{N}$ and $\delta=d_{\min}/V_{\text{pol}}$ with $d_{\min}$ a minimal

interpolymer's distance. Then the entire sequence of initiation times (or the possible polymerase positions) is coded as a fixed size binary vector $X=\left(x_1,\cdots,x_{N_{\max}}\right)$, where $N_{\max}=L/\delta$ with L the movie length. $x_i=1\left(1\le i\le N_{\max}\right)$ represents the ith position occupied by polymerase. The sum of X is the N_{pol}.

Third, we use the genetic algorithm to optimize X. The observed signal is denoted as $s_{\mathrm{obs}}(t)$. We will determine X by least-squares regression that minimizes the following objective function

$$\mathcal{O}(X)=\sum_{k=1}^{N_{frames}}\left(s(k\Delta;X)-s_{\mathrm{obs}}(k\Delta)\right)^2, \quad \text{(S118)}$$

where Δ is the movie time resolution and N_{frames} is the number of frames. Starting with a binary vector X_0 (pick N_{pol} positions to 1 and others to 0), we use a genetic algorithm to optimize the objective function. At each step, the genetic algorithm solver randomly selects parent individuals from a subpopulation and generates the next generation through recombination, crossover, and mutation. Over consecutive generations, the population maintains the optimal solution and 'evolves' towards it.

Finally, we perform local optimization by displacing the polymerase positions a few steps to the right or to the left based on the preliminary solution obtained by genetic algorithm, and further reduce the error between the reconstructed signal and the experimental signal.

Supplementary Figures

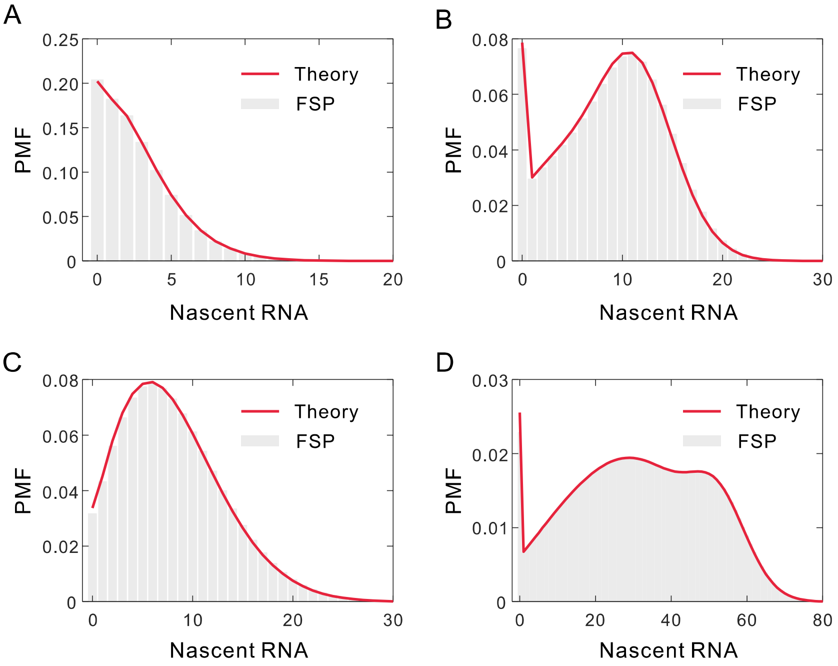

Figure S1. Comparison of steady-state nascent RNA distributions using analytical and numerical solutions. The analytical results (red solid curves) are computed using the exact expression of steady-state nascent RNA distribution, while the numerical results (histogram) are computed using the finite-state projection algorithm (FSP). (A)(B) Two representative distributions of nascent RNA for a three-state loop model. (C)(D) Two representative distributions of nascent RNA for a four-state loop model. The parameter values in the three-state loop models are set as: (A) $\lambda_{12} = \lambda_{23} = 0.03,\ \lambda_{31} = 0.05,\ \mu = 0.08,\ T = 150$; (B) $\lambda_{12} = \lambda_{23} = 0.1,\ \lambda_{31} = 0.02,\ \mu = 0.5,\ T = 25$. The parameter values in the four-state loop models are set as: (C) $\lambda_{12} = \lambda_{23} = \lambda_{34} = 0.8,\ \lambda_{41} = 0.4,\ \mu = 2,\ T = 10$; (D) $\lambda_{12} = \lambda_{23} = \lambda_{34} = 0.18,\ \lambda_{41} = 0.04,\ \mu = 1.8,\ T = 30$.

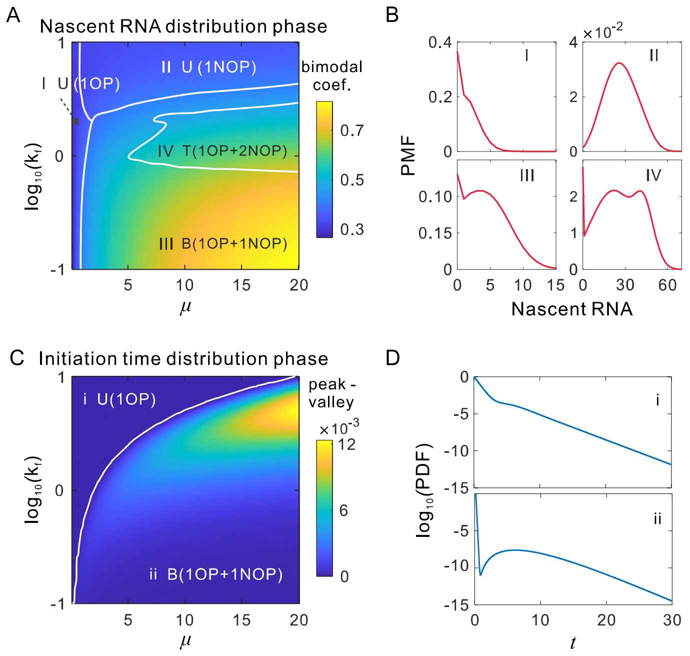

Figure S2. Stochastic bifurcation diagrams for steady-state nascent RNA distribution and initiation time distribution. (A) Diagram of steady-state nascent RNA distribution as a function of the initiation rate μ and the forward transition rate k_{f}, where the white curves classify the parameter space into four regions: I, II, III, and IV. Correspondingly, the nascent RNA distribution is classified into four types: U(1OP), U(1NOP), B(1OP+1NOP), and T(1OP+2NOP). (B) Four representative PMFs of nascent RNA for the four regions. (C) Diagram of initiation time distribution as a function of the initiation rate μ and the forward transition rate k_{f}, where the white curve classifies the parameter space into two regions: i and ii. Correspondingly, the initiation time distribution is classified into two types: U(1OP) and B(1OP+1NOP). (D) Two representative PDFs of nascent RNA for the two regions. In addition, the parameters are set as: $k_{\mathrm{b}} = 0.16 k_{\mathrm{f}}$, $T = 2.5$.

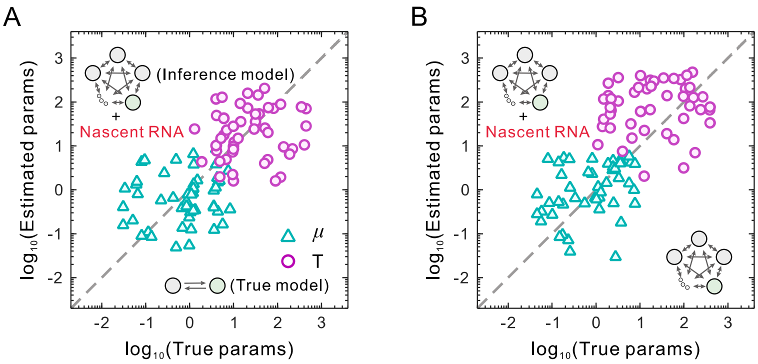

Figure S3. Parameter estimates using nascent RNA data from two-state and multi-state models, with inference performed using multi-state models. (A) Parameters estimated using multi-state models vs true parameters for datasets generated by two-state model. (B) Parameters estimated using multi-state models vs true parameters for datasets generated by multi-state models. In panels (A) through (B), the parameter sets are randomly generated from a large region of parameter space: $\lambda_{kl} \in \text{Uniform}(0.01,10)$, $\mu \in \text{Uniform}(0.01,10)$, $T \in \text{Uniform}(1,500)$.

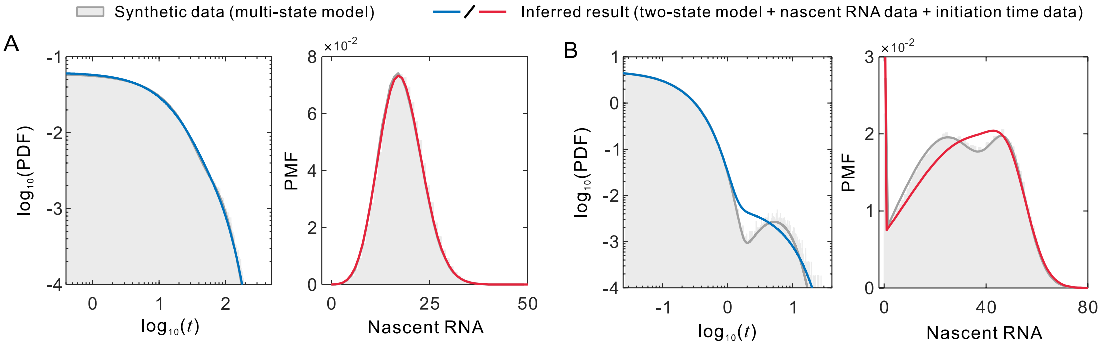

Figure S4. Comparison of the estimated distributions using two-state model with the actual distributions for synthetic data generated by multi-state models. (A) Shown is the illustration of the inferred distributions for initiation times and nascent RNA counts fitting well with the actual distributions. (B) Shown is the illustration of the inferred distributions for initiation times and nascent RNA counts fitting poor with the actual distributions. Here, we use five-state loop model to generate synthetic data, in which the forward transition rates are assumed to be the same denoted as k_{f}, and the backward transition rate is denoted as k_{b}. The used parameters for synthetic data production from the five-state loop model: $k_{\text{f}} = 0.09$, $k_{\text{b}} = 0.008$, $\mu = 0.06$, $T = 400$ in (A), and $k_{\text{f}} = 0.625$, $k_{\text{b}} = 0.1$, $\mu = 5$, $T = 10$ in (B). The inferred parameters using the two-state model: $k_{\text{on}} = 0.03786$, $k_{\text{off}} = 0.01551$, $\mu = 0.06247$, $T = 396.2935$ in (A), and $k_{\text{on}} = 0.2153$, $k_{\text{off}} = 0.1452$, $\mu = 5.0383$, $T = 10.2043$ in (B).

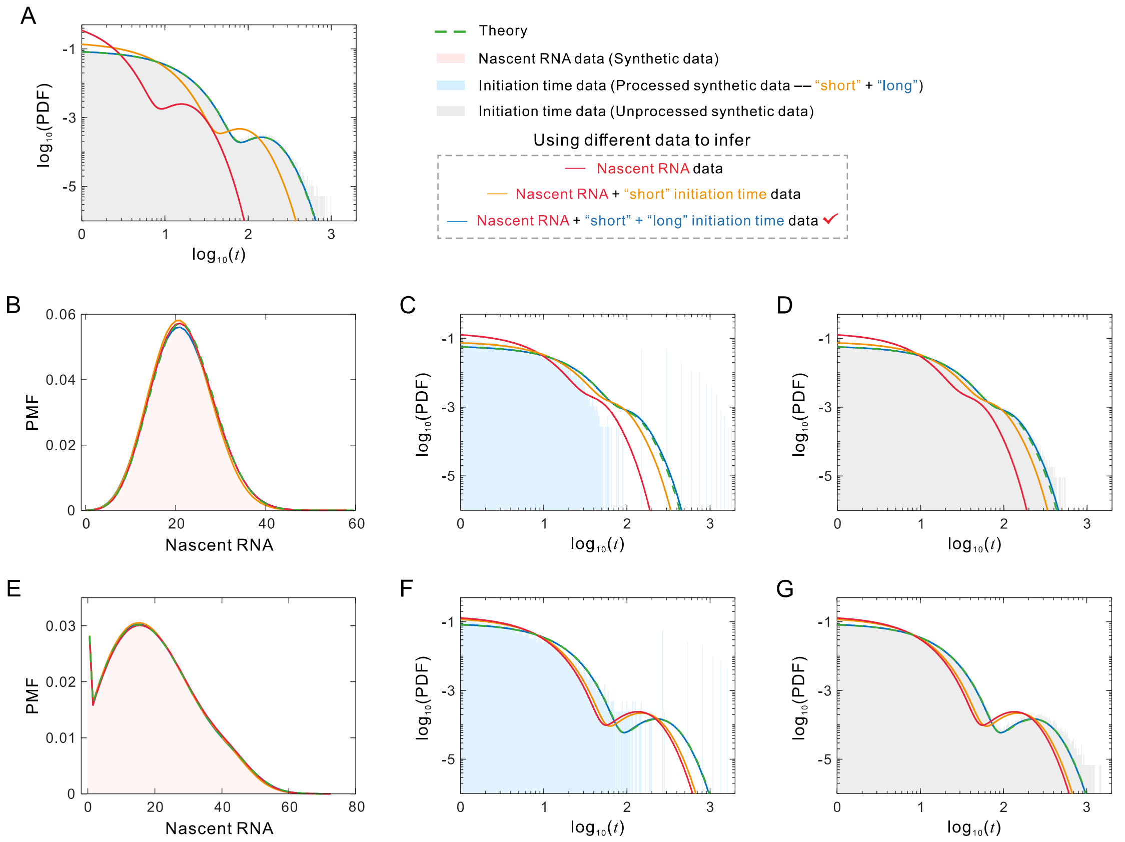

Figure S5. Testing the accuracy of theory and inference methods using synthetic imaging data. (A) Shown are the estimated initiation-time distributions using three inference methods, compared with the true initiation-time distribution based on theory, and the histogram of initiation time distribution using unprocessed simulated data, where the inferences from nascent RNA data and the integrated short and long initiation time data fit the histogram well, whereas the other two inferences show significant differences from the histogram. (B)–(D) Shown are illustrations testing the accuracy of the proposed theory and inference methods, compared with the other two inference methods, where the true nascent RNA and initiation distributions are both unimodal. (E)–(F) Shown are illustrations testing the accuracy of the proposed theory and inference methods, compared with the other two inference methods, where the true nascent RNA and initiation distributions are both bimodal. The parameters used for the model produced data and the estimated parameters from three inferred methods are listed in Table S1.

Supplementary Tables

Table S1: Comparison of parameters used in the models with those estimated from nascent RNA data (Method I), nascent RNA data plus short initiation data (Method II), and nascent RNA data plus integrated short and long initiation time data (Method III). In the five-state loop model, the forward transition rates are assumed to be the same denoted as k_{f}, and the backward transition rate is denoted as k_{b}.

Fig. 4C-D and Fig. S5A	Parameters		$k_{\mathrm{f}}\,[s^{-1}]$	$k_{\mathrm{b}}\,[s^{-1}]$	$\mu\,[s^{-1}]$	$T\,[s]$
	Model used parameters		0.025	0.005	0.09	500
	Estimated parameters	Method I	0.2273	0.04516	0.8113	54.6782
		Method II	0.04534	0.008482	0.16122	273.4949
		Method III	0.02456	0.005069	0.09067	500.9434
Fig. S5B-D	Parameters		$k_{\mathrm{f}}\,[s^{-1}]$	$k_{\mathrm{b}}\,[s^{-1}]$	$\mu\,[s^{-1}]$	$T\,[s]$
	Model used parameters		0.05	0.005	0.06	500
	Estimated parameters	Method I	0.07138	0.008422	0.08084	392.2988
		Method II	0.125	0.01206	0.1487	198.4448
		Method III	0.04838	0.005552	0.05993	522.127
Fig. S5E-G	Parameters		$k_{\mathrm{f}}\,[s^{-1}]$	$k_{\mathrm{b}}\,[s^{-1}]$	$\mu\,[s^{-1}]$	$T\,[s]$
	Model used parameters		0.0145	0.0045	0.09	500
	Estimated parameters	Method I	0.02461	0.007327	0.15093	292.2159
		Method II	0.02231	0.006665	0.135	325.2078
		Method III	0.01473	0.004592	0.09123	493.3517

Table S2: The parameters inferred from the integration of nascent RNA data and short and long initiation data for three cell lines using two-state and multi-state loop models. In the multi-state loop models, the forward transition rates are assumed to be the same denoted as k_f, and the backward transition rate is denoted as k_b. In addition, MS represents the model score, which is a standardized measure derived from the maximum likelihood values. The highest MS indicates the best-fitting model.

No Tat	Inferred models		2-state	3-state	4-state	5-state
	Estimated Parameters	$k_f\,[s^{-1}]$	0.001439	0.002244	0.003141	0.004247
		$k_b\,[s^{-1}]$	0.02333	0.01517	0.0125	0.01117
		$\mu\,[s^{-1}]$	0.07664	0.06625	0.06192	0.05996
		$T\,[s]$	1411.035	1373.9135	1314.9577	1208.792
	Model scores	MS	2120.16	4885.86	1565.19	797.626
Low Tat	Inferred models		2-state	3-state	4-state	5-state
	Estimated Parameters	$k_f\,[s^{-1}]$	0.001845	0.00308	0.004472	0.006085
		$k_b\,[s^{-1}]$	0.02099	0.0138	0.01146	0.01022
		$\mu\,[s^{-1}]$	0.1003	0.0909	0.08752	0.0859
		$T\,[s]$	1382.9765	1226.3375	1106.4199	1004.7336
	Model scores	MS	6201.11	1143.43	492.391	307.541
High Tat	Inferred models		2-state	3-state	4-state	5-state
	Estimated Parameters	$k_f\,[s^{-1}]$	0.008666	0.01287	0.01704	0.02133
		$k_b\,[s^{-1}]$	0.02582	0.01544	0.01232	0.01091
		$\mu\,[s^{-1}]$	0.1956	0.177	0.16976	0.1668
		$T\,[s]$	456.5929	433.7675	420.0765	407.0278
	Model scores	MS	515.297	366.288	299.027	258.315

Table S3: The parameters inferred from nascent RNA data for three cell lines using two-state and multi-state loop models. In the multi-state loop models, the forward transition rates are assumed to be the same denoted as k_{f}, and the backward transition rate is denoted as k_{b}. In addition, MS represents the model score, which is a standardized measure derived from the maximum likelihood values. The highest MS indicates the best-fitting model.

	Inferred models		2-state	3-state	4-state	5-state
No Tat	Estimated Parameters	$k_{\mathrm{f}}\,[s^{-1}]$	0.005438	0.007321	0.006479	0.007098
		$k_{\mathrm{b}}\,[s^{-1}]$	3.2144	2.6145	1.7508	1.3417
		$\mu\,[s^{-1}]$	8.9511	9.6839	7.3554	6.01862
		$T\,[s]$	408.4791	453.8287	681.3152	783.5323
	Model scores	MS	114.853	174.836	233.434	291.111
	Inferred models		2-state	3-state	4-state	5-state
Low Tat	Estimated Parameters	$k_{\mathrm{f}}\,[s^{-1}]$	0.002117	0.0075167	0.007435	0.01735
		$k_{\mathrm{b}}\,[s^{-1}]$	1.004671	0.5273	0.6785	0.7182
		$\mu\,[s^{-1}]$	4.2683	2.8542	4.2496	4.8277
		$T\,[s]$	2962.4512	545.5546	720.405	384.1039
	Model scores	MS	176.014	225.554	268.168	291.153
	Inferred models		2-state	3-state	4-state	5-state
High Tat	Estimated Parameters	$k_{\mathrm{f}}\,[s^{-1}]$	0.07298	0.05076	0.1129	0.1431
		$k_{\mathrm{b}}\,[s^{-1}]$	0.3184	0.07119	0.08604	0.0891
		$\mu\,[s^{-1}]$	2.0362	0.7474	1.1382	1.2127
		$T\,[s]$	58.8695	114.203	65.0312	63.204
	Model scores	MS	474.363	459.481	433.895	381.344

Supplementary References

[1] Weispfenning V. Quantifier elimination for real algebra—the cubic case. in *Proceedings of the International Symposium on Symbolic and Algebraic Computation*. **1994**, 258-263

[2] Yang L. Recent advances on determining the number of real roots of parametric polynomials. *J Symb Comput*. 1999; 28:225-242.

[3] Cox D. *Renewal Theory.* **1967**. Methuen, London.

[4] Nicoll AG, Szavits-Nossan J, Evans MR,Grima R. Transient power-law behaviour following induction distinguishes between competing models of stochastic gene expression. *bioRxiv*. 2023:2023.12. 30.573521.

[5] Tantale K, Garcia-Oliver E, Robert M-C, L'hostis A, Yang Y, Tsanov N, Topno R, Gostan T, Kozulic-Pirher A,Basu-Shrivastava M. Stochastic pausing at latent HIV-1 promoters generates transcriptional bursting. *Nat Commun*. 2021; 12(1):4503.
